# Adaptive Control-based frequency control strategy for PV/ DEG/ battery power system during islanding conditions

**DOI:** 10.1038/s41598-025-19341-8

**Published:** 2025-11-18

**Authors:** Mohamed A. Ghalib, M. S. Elbrolsy, R. M. Mostafa, H.E. Keshta

**Affiliations:** 1 Process Control Technology Department, Faculty of Technology and Education, Beni-Suef University, Beni-Suef, Egypt; 2 Electrical Engineering Department, Faculty of Engineering at Shoubra, Benha University, Benha, Egypt

**Keywords:** Photovoltaics, Microgrids, Frequency control, Diesel engine generators, Fuzzy logic controllers, Renewable energy sources., Engineering, Electrical and electronic engineering

## Abstract

The concept of Islanded Hybrid Power System (IHPS) has attracted considerable interest lately, especially for energizing remote or energy-poor locations. IHPS are more dependable and cost-effective alternatives to systems using only one energy source when properly constructed. IHPS configuration, including Diesel Engine Generator (DEG), Photovoltaic (PV) systems, and Battery Storage (BATT) elements, are desirable for islanded systems about price and dependability. IHPS mostly use Renewable Energy Sources (RES) for power production, which is variable. Consequently, these variations often make it difficult for traditional control systems to maximize efficiency across various operating environments. The current research discusses the requirement for more effective frequency control in IHPS by suggesting a Model Reference Adaptive Control-Fuzzy Proportional Integral based Whale Optimization Algorithm (MRAC-FPI-WOA) controller. The proposed controller can efficiently manage a range of disturbances by dynamically adjusting its control techniques. The current research conducts an evaluation study comparing the effectiveness of the suggested MRAC-FPI-WOA controller against FPI-WOA, PI-WOA, and PI-PSO controllers. The key evaluation criteria are the ability to maintain stability in frequency within the IHPS and the effectiveness of power production in the overall system. The results demonstrate the superior performance of the MRAC-FPI-WOA controller across diverse operational scenarios. Notably, during a three-phase fault at Bus2, the MRAC-FPI-WOA controller achieves significant performance enhancements over the PI-PSO controller, with reductions of 59.05% in maximum overshoot (%$\:{\text{M}}_{\text{p}}$), 72.83% in maximum undershoot (%$\:{\text{M}}_{\text{u}\text{s}}$), 32.07% in settling time ($\:{\text{T}}_{\text{s}}$), and 34.81% in the integral of time-weighted absolute error (ITAE). A similar trend is observed during a three-phase fault at the tie-line, where the MRAC-FPI-WOA controller yields improvements of 57.47% in %$\:{\text{M}}_{\text{p}}$, 79.36% in %$\:{\text{M}}_{\text{u}\text{s}}$, 40.9% in $\:{\text{T}}_{\text{s}}$, and 78.08% in ITAE. Furthermore, the controller exhibits exceptional dynamic responsiveness to ramp variations in solar radiation, substantially reducing %$\:{\text{M}}_{\text{p}}\:$by 96.72%, %$\:{\text{M}}_{\text{u}\text{s}}$ by 95.24%, $\:{\text{T}}_{\text{s}}\:$by 22.79%, and ITAE by 89.69%. Additionally, it demonstrates robust adaptability to random solar radiation fluctuations, consistently optimizing transient response with reductions of 96.63% in %$\:{\text{M}}_{\text{p}}$, 99.58% in %$\:{\text{M}}_{\text{u}\text{s}}$, 22.07% in $\:{\text{T}}_{\text{s}}$, and 95.23% in ITAE.

## Introduction

Sustainable energy solutions are being widely adopted in modern power systems to reduce environmental impact and enhance grid performance. While they improve efficiency, voltage stability, and ecological benefits, their excessive integration can challenge grid operation, protection, and control^1^. A microgrid (MG) represents a localized power network that integrates renewable generation sources (e.g., photovoltaic arrays, wind turbines) with energy storage components (e.g., battery banks) to form a self-contained electrical system^2^. Hybrid Power System (HPS) operation can switch between two key modes: independent (islanded) and grid-tied operation. IHPS are considered the most effective approach for supplying electricity to remote and rural areas due to their technical feasibility and cost-efficiency^3^. The intermittent and unpredictable nature of RES in HPS can cause voltage instability and oscillations, potentially affecting connected loads. To ensure system reliability and the quality of electrical supply, an effective control strategy must be developed, allowing the HPS to operate efficiently despite uncertainties in weather conditions and load variations during the system runs in real-time^4^. As a result, IHPS operations necessitate BATT to retain surplus energy generated by the HPS, ensuring power availability when production is insufficient to meet demand. This study examines the dynamic performance of IHPS under various operating conditions.

The efficient control and management of HPS require advanced strategies and algorithms to optimize the utilization of RES, manage BATT, and ensure a stable and reliable power supply^5^. One of the most critical aspects of HPS operation is frequency stability, which is essential for maintaining high-quality electricity for connected loads. Fluctuations in frequency arise from variations in power generation and consumption, highlighting the necessity for robust frequency regulation mechanisms to maintain HPS stability and performance^6^. Several approaches can be applied to frequency regulation in IHPS. One widely used method involves BATT to compensate for fluctuations in RES generation, ensuring a steady and secure system frequency. Other techniques include advanced control strategies and demand-side management approaches. Extensive research has explored various control methodologies for regulating the operation of standalone hybrid MG^7^. A control strategy proposed in^8^ focuses on biogas-based MG, allowing the system to increase or decrease power generation in response to disturbances caused by fluctuations in RES input or load demand. However, a key drawback of this approach is its inability to respond swiftly to sudden changes, potentially leading to transient instability. Additionally, the controller may lack robust fault detection and isolation capabilities, and its effectiveness could decline when scaling up or integrating with larger power grids. To enhance frequency regulation and stability, Ref^9^ suggests using an adaptive active power droop controller along with voltage setpoint adjustment in IHPS. These control mechanisms aim to improve the overall performance of HPS systems. Furthermore, Ref^10^ explores a control technique for BATT designed to mitigate frequency variations and enhance the dynamic response of IHPS. To achieve superior frequency stability during transient disturbances, they propose the use of a Piecewise Linear-Elliptic (PLE) droop characteristic in BATT control systems. This control characteristic enables a faster equilibrium between consumption and power generation, leading to improved frequency regulation in HPS. However, while the PLE controller can reduce frequency variations, it does not fully eliminate them. Additionally, it may be less effective when load demand is lower than power generation, potentially causing sudden fluctuations in BATT output power. In^11^, a voltage regulation strategy for IHPS incorporating PV and BATT was examined. Ref^12^ deals with the control of the Vehicle Cruise Control System (VCCS) based on a Model Predictive Controller (MPC) in parallel with the conventional PID controller. The study evaluates the technique’s effectiveness in improving HPS performance, but it does not fully address key challenges related to islanded mode regulations, frequency stability, protection settings, power management, and load diversity handling in HPS.

Optimization algorithms inspired by biological and natural phenomena are classified as metaheuristic approaches. Unlike traditional mathematical optimization techniques, which often struggle with complex search spaces, metaheuristic algorithms effectively explore potential solutions to high-dimensional, nonlinear, and multi-modal optimization problems. As a result, techniques like the WOA, Particle Swarm Optimization (PSO), and Genetic Algorithm (GA) have gained widespread attention across various fields. These techniques are commonly used to optimize system performance by fine-tuning control parameters in advanced control systems, including Proportional-Integral (PI), Proportional-Integral-Derivative (PID), Fuzzy Proportional-Integral (FPI), Fractional-Order PI (FOPI), and Fuzzy-Fractional Order PID (FFOPID) controllers.

Recent studies highlight innovative bio-inspired optimization techniques for power systems. In^13^ Bio-Dynamic Grasshopper Optimization Algorithm (BDGOA) is used to optimize Tilt-Derivative with N-filter plus PI controllers for frequency/tie-line oscillation damping. In^14^ Diligent Crow Search Algorithm (DCSA) is employed for solar cell parameter identification to maximize PV output. In^15^ Hybrid Adaptive Ant Lion Optimization (HAALO) with PI/FOPID controllers is developed to enhance Switched Reluctance Motor performance through adaptive mutation and torque ripple reduction. In^16^ BDGOA is applied for precise parameter estimation across five solar module technologies. In^17^ Crow-Search Algorithm (CSA) is employed to optimize Type-2 Fuzzy Cascade (T2F-CPIF) controllers for robust frequency/tie-line error mitigation in hybrid systems under contingency scenarios. In^18^ WOA is utilized to enhance Fuzzy Cascade PD-PI controllers, substantially improving microgrid transient response during operational disturbances. For secondary frequency regulation. In^19^ Improved Salp Swarm Optimization (I-SSO) tuned Type-II Fuzzy PID controller is implemented to maintain nominal frequency and tie-line power despite uncertainties. Complementing these approaches. In^20^ advanced Sine Cosine Algorithm (a-SCA) is implemented to optimize the Fractional-Order Fuzzy for precise generation-demand balancing in fluctuating conditions. In^21^ Coati Optimization Algorithm (COA) was implemented to optimize the parameters of Fuzzy-PI (FPI) and conventional PI controllers, significantly improving the frequency regulation performance in a two-area power system. In^22^ modified Sea-horse Optimization (SHO) method is developed for tuning Proportional-Integral-Derivative-Tilt (PID-T) controllers in renewable-integrated multi-area systems. In^23^ SHO is enhanced to optimize Model Predictive Control (MPC), PID, Fractional order proportional integral derivative (FOPID), and Tilted Integral Derivative (TID) controllers for complex power networks. For cyber-resilient operation. In^24^ Chaos Quasi-Oppositional SHO (CQOSHO) proposes to tune a novel Cascaded tilted-FO derivative with filter ($\:{\text{C}\text{P}\text{D}}^{{\upmu\:}}$F − TI) controller with deep learning capabilities. Complementing these advances. In^25^ Opposition-based SHO (OSHO) is developed for hybrid systems, optimizing TID-MPC controllers to manage renewable penetration and virtual inertia challenges. In^26^ Dragonfly Search Algorithm (DSA) is employed to optimize an Adaptive Fractional Order PI (AFOPI) controller for precise motor speed regulation. In^27^ DSA is utilized for tuning a novel cascaded PI-(FOP + PD) structure to mitigate frequency fluctuations in power systems. Complementing these approaches. In^28^ Tunicate Search Algorithm (TSA) is implemented to enhance transient stability in hybrid grids through optimized Tilt Fractional Order PID (TFOPID) control. These developments showcase the effectiveness of bio-inspired optimization in addressing diverse control challenges across electromechanical and power system applications.

The proposed WOA has demonstrated remarkable efficacy across diverse domains, especially in enhancing control system configurations^29^. For instance, when applied to PID controllers, WOA-optimized systems achieve rapid transient responses, minimized steady-state deviations, and enhanced oscillation damping in contemporary power grids, outperforming GA and Artificial Bee Colony (ABC) approaches^30^. In renewable energy applications, WOA-driven Fractional-Order Proportional-Integral Controllers (FOPIλ) excel within sensor-free speed control applications for solar-fed permanent-magnet brushless DC motors. These systems surpass Bat Algorithm (BA) and Grey Wolf Optimizer (GWO) implementations by reducing tracking errors and shortening convergence intervals^31^. Similarly, WOA-enhanced FFOPID controllers integrated into active vehicle suspension models significantly attenuate driver vibrations relative to Fractional-Order PID (FOPID) and PSO-tuned counterparts^32^. Furthermore, WOA-based Maximum Power Point Tracking (MPPT) techniques applied to Proton Exchange Membrane Fuel Cells (PEMFC) dynamically adapt to electrolyte hydration fluctuations, securing optimal power extraction with greater efficiency than Perturb-and-Observe (P&O), Fuzzy Logic Controller (FLC), and PSO methodologies^33^. These advancements underscore WOA’s versatility in resolving nonlinear, multi-variable challenges across energy and mechanical systems.

The Research gap of this study includes:

- Limitations of Traditional Controllers: Existing IHPS studies rely on PI, PID, FOPI, and FPI controllers, which face challenges in handling nonlinear system dynamics and severe grid disturbances. These controllers show slow transient recovery, increased frequency overshoot, and prolonged settling times, compromising system stability.
- Inadequate Handling of Diverse Disturbances: Prior research does not sufficiently address the combined impact of gradual fluctuations (e.g., solar irradiance) and severe grid anomalies (e.g., three-phase faults, load shedding), causing instability in IHPS.
- Lack of Adaptive Frequency Control: Many existing controllers do not adapt to varying renewable energy fluctuations and load changes, leading to poor frequency regulation and reduced system efficiency.
- Deficiencies in Power Coordination and Scalability: Conventional methods do not effectively coordinate power generation, storage, and demand, limiting overall system reliability and scalability for real-world applications.
- Underutilization of Intelligent Optimization in Control Tuning: Automated gain calibration for frequency controllers is underdeveloped, and no framework integrates nonlinear adaptive control with swarm-based optimization for dynamic tuning.
- Need for an Advanced Control Strategy: A novel approach is crucial for optimizing frequency regulation, transient stability, and operational robustness in IHPS. The integration of MRAC-FPI-WOA gives a promising answer by enabling adaptive tuning in real time and intelligent power coordination in IHPS.

The contributions of this study include:

(i) Methodological innovations:

- Investigates the transient behavior and operational robustness of integrated PV-BATT-DEG power systems under both gradual environmental perturbations (e.g., incremental solar irradiance shifts) and severe grid anomalies (e.g., three-phase faults, abrupt load shedding).

- Proposes a load frequency control to synchronize power generation, storage, and demand in IHPS. This strategy strengthens inter-component coordination, adapts to real-time grid dynamics, and ensures voltage/frequency stability during fluctuating renewable outputs and load transitions.
- Develops a non-linear adaptive controller (MRAC-FPI-WOA). This innovation optimizes transient frequency recovery across diverse operating regimes, outperforming PI-PS0, PI-WOA and FPI-WOA controllers in damping oscillations and minimizing settling times.
- Enhances the technical feasibility of large-scale renewable adoption by mitigating frequency volatility in IHPS. This advancement aligns with global sustainability agendas, reducing fossil dependency while improving energy distribution reliability in decentralized grids.

(ii) Algorithmic implementations:

- Proposes Beta-based MPPT technique, which enhances the tracking accuracy and dynamic performance of the PV system by adaptively controlling power extraction based on a novel intermediary variable (β), rather than relying solely on conventional power change methods. The WOA is integrated with the beta-based MPPT controller to enhance the total efficiency of the PV system.

- Leverages the PSO and WOA to automate gain calibration for proposed controllers. WOA effectively resolves nonlinearities and component interdependencies, ensuring the best dynamic response in variable operating conditions.

(iii) Simulation/experimental findings:

- Demonstrates the MRAC-FPI-WOA’s superiority through rigorous metrics: lower maximum overshoot (%$\:{\text{M}}_{\text{p}}$), and trough undershoot (%$\:{\text{M}}_{\text{u}\text{s}}$) at lower frequencies, faster settling time ($\:{\text{T}}_{\text{s}}$), and a decrease in the integral of time-weighted absolute error (ITAE) in contrast to benchmarks. These results validate its capability to sustain grid stability during both minor and catastrophic disturbances.

This paper’s remaining sections are arranged as follows: This paper systematically explores the design and control of IHPS components PV systems, DEG, and BATT in "Modeling of islanded hybrid power system", proceeding to evaluate four frequency control strategies, including MRAC-FPI-WOA, FPI-WOA, PI-WOA, and PI-PSO controllers in "Frequency Control". A detailed simulation-based analysis in "Results and discussion" compares controller performance under seven scenarios, including three-phase faults, step/ramp/random solar irradiance fluctuations, as well as abrupt load changes and composite disturbances. Cases 3 (step irradiance) and 6 (sudden load shift) are tested concurrently to assess robustness under hybrid stresses. The study concludes in "Conclusions" that the MRAC-FPI-WOA controller, enhanced by metaheuristic tuning, outperforms conventional methods in maintaining frequency stability and power quality across all disturbances, underscoring its potential to enhance HPS resilience in real-world applications characterized by renewable intermittency and operational uncertainties.

## Modeling of islanded hybrid power system

**Fig. 1:**
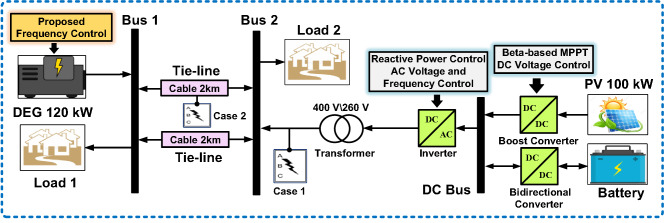
Block diagram of the proposed IHPS.

This research undertaking centers its analytical scope on the architectural design and functional dynamics of Alternating current (AC) IHPS, integrating multiple distributed energy resources, including DEG, PV, AC consumer loads, and advanced BATT solutions. Figure 1 delivers a refined schematic overview of the IHPS infrastructure, emphasizing the interconnection of the DEG to the primary AC distribution backbone through sophisticated power electronic interfaces. These components perform dual critical functions: harmonizing the phase and frequency characteristics of disparate AC power sources while enabling efficient conversion of Direct Current (DC) electricity harvested from solar panels into HPS-compatible alternating current waveforms. The BATT incorporates a bidirectional power conversion apparatus, engineered to transition seamlessly between AC-to-DC operational modes during energy accumulation cycles and DC-to-AC modes during discharge phases. This dual functionality not only stabilizes the HPS against voltage fluctuations and transient load imbalances but also enhances operational flexibility during system upkeep or component servicing. This comprehensive framework underscores HPS’s resilience in maintaining uninterrupted power delivery while accommodating diverse energy inputs and dynamic load profiles.

### Modeling of PV system

**Fig. 2:**
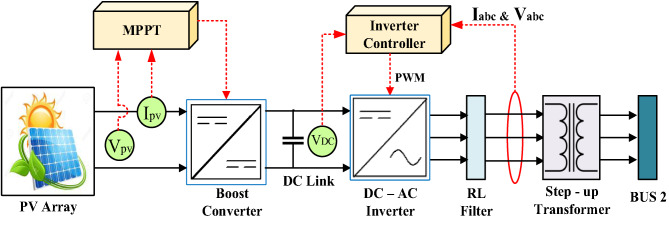
Schematic of a Solar PV System.

This section introduces a detailed and robust simulation framework designed to be a high-performance PV system. The system architecture encompasses several critical elements: a 100-kilowatt solar panel array, a step-up DC-DC converter, a power inversion unit, and a voltage adjustment transformer. A methodically structured schematic diagram and computational model, illustrated in Fig. 2, offer a comprehensive and logically organized visualization of the entire configuration. Sunlight is harvested by a solar array and converted into DC electricity. To enable compatibility with standard power distribution networks, this DC output must undergo conversion to AC. This critical transition is eased by the inverter module, which transforms the unidirectional electrical flow into a three-phase AC output synchronized with grid specifications. Subsequently, a voltage-elevating transformer amplifies the AC voltage to match the grid’s operational requirements, ensuring seamless energy transfer.

Each component operates synergistically: the Boost converter optimizes the DC voltage from the solar panels to maximize efficiency, the inverter ensures waveform compatibility with HPS standards, and the transformer bridges voltage disparities to enable stable power injection. This integrated approach highlights the system’s capability to efficiently harness, process, and deliver renewable energy while adhering to technical and operational benchmarks for grid integration^34,35^.

#### Modeling of PV cell

**Fig. 3:**
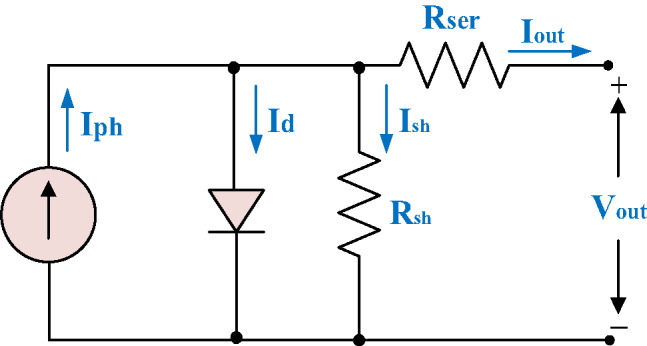
Schematic representation of a basic diode-based model used for PV solar cells.

Various mathematical representations describing the functionality and efficiency of solar panels have been extensively documented in previous studies. For real-time simulation, it is necessary to develop an equivalent circuit model of PV cells. Among the different approaches, the single-diode model is the most widely adopted by researchers. This circuit configuration comprises, at a minimum, four key elements: a photocurrent source ($\:{I}_{ph}$), a diode (*D*), a shunt resistance ($\:{R}_{sh}$), and a series of resistance ($\:{R}_{ser}$). Based on the equivalent single-diode model of a PV cell depicted in Fig. 3, the output current ($\:{I}_{out}$) can be expressed mathematically in the following way^36,37^.

1 $$\:{I}_{out}\text{\:=\:}{I}_{ph}{N}_{P}-{I}_{rs}{N}_{P}\text{}\left[{e}^{\frac{q({V}_{{out}}+\:{R}_{{ser}}{I}_{{out}})}{{AKT}{N}_{S}}}-1\right]-{N}_{P}\text{}\left[q\left(\:\frac{\:\left({V}_{out}+\:{R}_{ser}{I}_{out}\right)}{{{R}_{sh}N}_{S}}\:\right)\right]$$

2 $$\:{I}_{ph}\text{\:=\:}\left[{I}_{sc}+{k}_{i}(\:T-{T}_{r}\:)\right]\:\left(\frac{E}{1000}\right)\text{}$$

Where$\:\:\left({N}_{P}\right)$ is the number of PV cells arranged in parallel, ($\:{I}_{rs})$ is The PV cell’s reverse leakage current, (*q*) is the electric charge of an electron,$\:{(V}_{out})$ is the cell’s output voltage, (*A*) is the diode ideality factor, (*K*) is the Boltzmann constant, (*T*) is the temperature measured in Kelvin, $\:{(N}_{S})$ is the total PV cells wired in a series connection,$\:{\:(\text{I}}_{\text{s}\text{c}})$ is the short-circuit current, $\:{(k}_{i})$ is the short circuit current factor, $\:\left({T}_{r}\right)$ is the cell reference temperature and (*E*) is the solar irradiance.

**Fig. 4:**
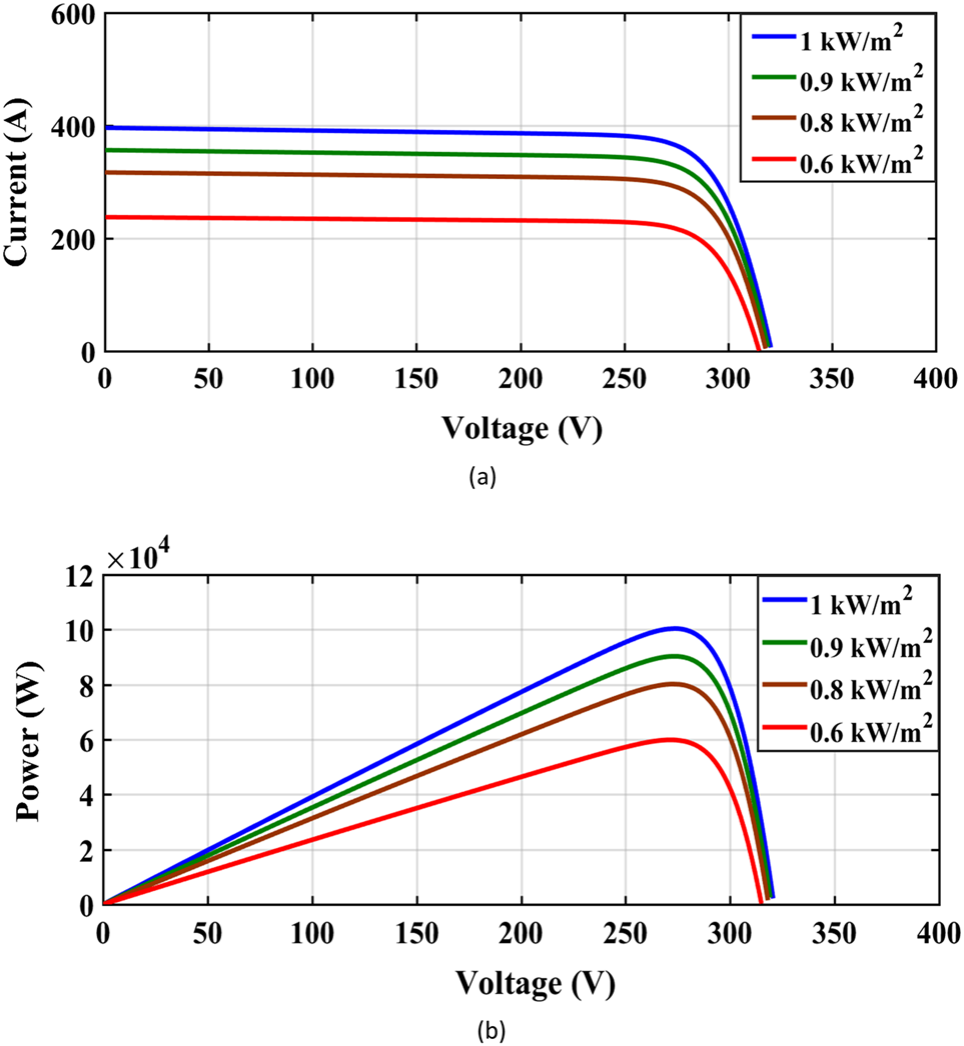
(**a**) I-V curve and (**b**) P-V characteristics of solar cells at varying irradiation levels.

Figure 4(a) and Fig. 4(b) depict the I-V and P-V characteristics of the PV cell, derived from a MATLAB-based computational model. These findings provide critical insights into the operational dynamics of the solar module under fluctuating irradiance scenarios, revealing how variations in solar intensity influence electrical output characteristics such as Maximum Power Point (MPP), open-circuit voltage $\:{(V}_{oc}$, and $\:{I}_{sc})$. The simulations show the nonlinear relationship between irradiance levels and energy conversion efficiency, emphasizing the importance of adaptive control strategies for optimizing solar harvesting in real-world environmental conditions.

#### Modeling of boost converter

**Fig. 5:**
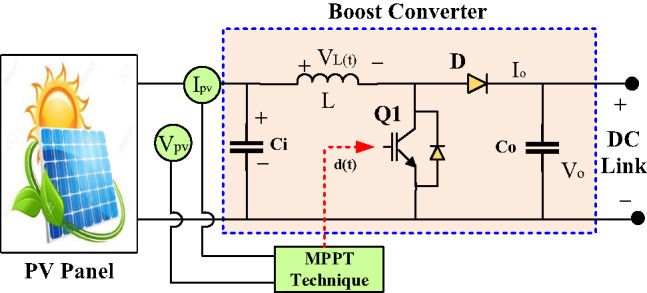
Circuit diagram of a boost converter.

A basic DC-DC boost converter is employed to deliver power from the PV to the DC link and the inverter once the matching condition between them is met. This matching is achieved by applying a suitable duty cycle (ranging between 0 and 1). The converter’s switching element, typically an IGBT, is regulated using a PWM signal. Figure 5 displays the Simulink model layout of the boost converter. The mathematical relationships governing the converter’s input and output parameters are expressed through the following Eqs^35,36^.

3 $${\text{~~}}{V_{o{\text{~~}}}}=~{V_{pv{\text{~~}}}}/1 - D$$

4 $${\text{~~}}{I_{o{\text{~~}}}}={\text{~~}}{I_{in{\text{~~}}}}\left( {1 - D} \right)$$

Here, the input and output voltages, along with the duty cycle, are represented as$\:{\:\:(V}_{o\:}$, $\:{V}_{in}$, and *D)*, respectively. The roles of the boost converter’s inductor (*L*) and capacitor (*C*) elements are specified as follows^35,36^:

5 $$L={V_{pv{\text{~~}}}}\left( {{V_{o{\text{~~}}}} - {V_{pv{\text{~~}}}}} \right)/f\left( {\Delta I} \right){V_{o{\text{~~}}}}$$

6 $$C={I_{o{\text{~~}}}}\left( {{V_{o{\text{~~}}}} - {V_{pv{\text{~~}}}}} \right)/f\left( {\Delta V} \right){V_{o{\text{~~}}}}$$

Where ($\:f$) is the frequency, $\:(\varDelta\:I$ and $\:\varDelta\:V)$ are the current and voltage ripple.

#### Beta-based MPPT technique

The β-MPPT method involves observing an intermediate variable called ($\:\beta\:$), rather than directly tracking power variations, as outlined in Eqs. (7) and (8)^36,37^.

7 $$\beta =ln\left( {\frac{{{I_{pv}}}}{{{V_{pv}}}}} \right) - C \times {V_{pv}}$$

8 $$C=\frac{q}{{NAKT}}$$

Here, $\:\left({I}_{pv}\right)$ is the output current, (*C)* is the diode constant, and (N) is the total count of solar cells contained in the module.

**Fig. 6:**
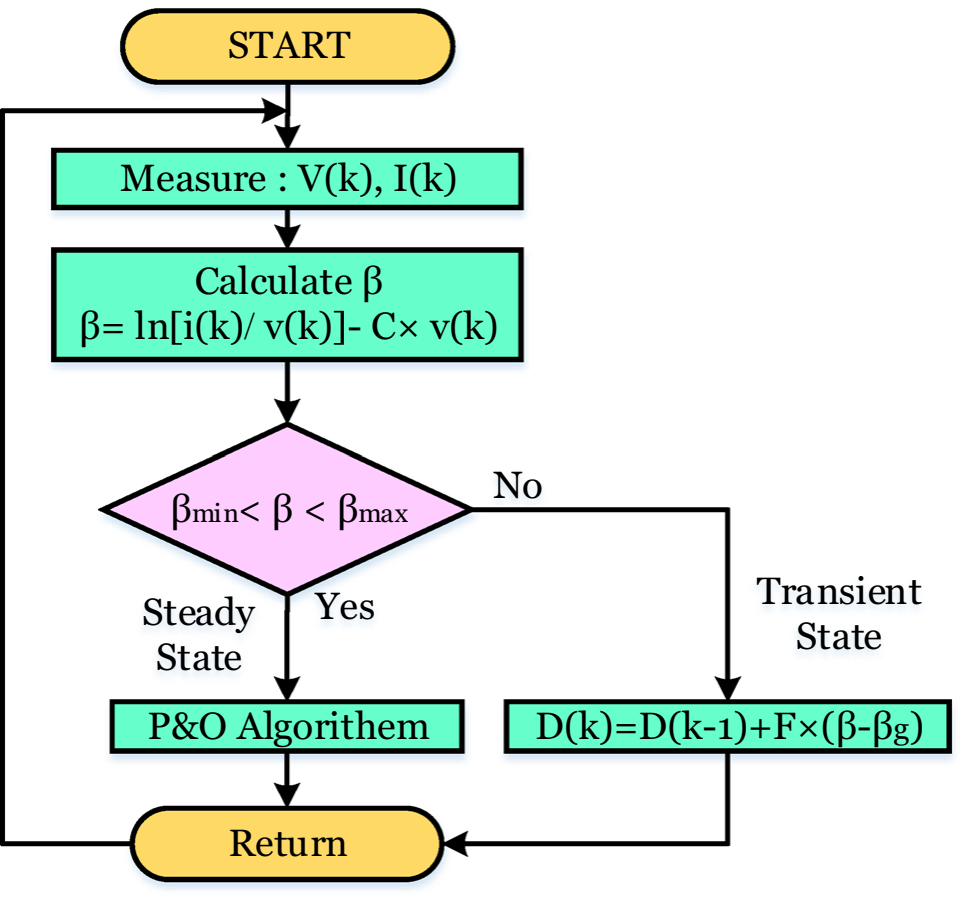
Flow chart of β-MPPT.

This method uses a hybrid step-size strategy, applying a variable step during dynamic changes and a fixed step during stable operating conditions. As outlined in Fig. 6, the algorithm begins by continuously observing voltage and current values to compute the intermediary beta parameter. If the calculated beta lies within a designated threshold range ($\:{\beta\:}_{min}$ to $\:{\beta\:}_{max}$), the system is in a steady state, and a fixed step is applied. If beta falls outside this range, the algorithm identifies a transient phase and switches to a P&O approach. In this stage, the variable step size, denoted as ΔD, is adjusted based on a reference parameter called $\:{(\beta\:}_{g})$, which is defined mathematically in Eq. (9)^36,37^.

9 $$\Delta D=F \times \left( {\beta - {\beta _g}} \right)$$

Where (F) is the scaling factor.

The WOA discussed in Sect. 4 is integrated with the beta-based MPPT controller to enhance the total efficiency of the PV system. Within this hybrid framework, the scaling factor (*F*) is essential for adaptively regulating the step size (*∆D*) during the dynamic response phase of the Beta MPPT method. Selecting the perfect value for (*F*) is key to achieving:

- Rapid tracking of the Maximum PowerPoint (MPP).
- Minimized fluctuations during steady-state operation.
- Enhanced performance across various levels of sunlight and temperature conditions.

**Fig. 7:**
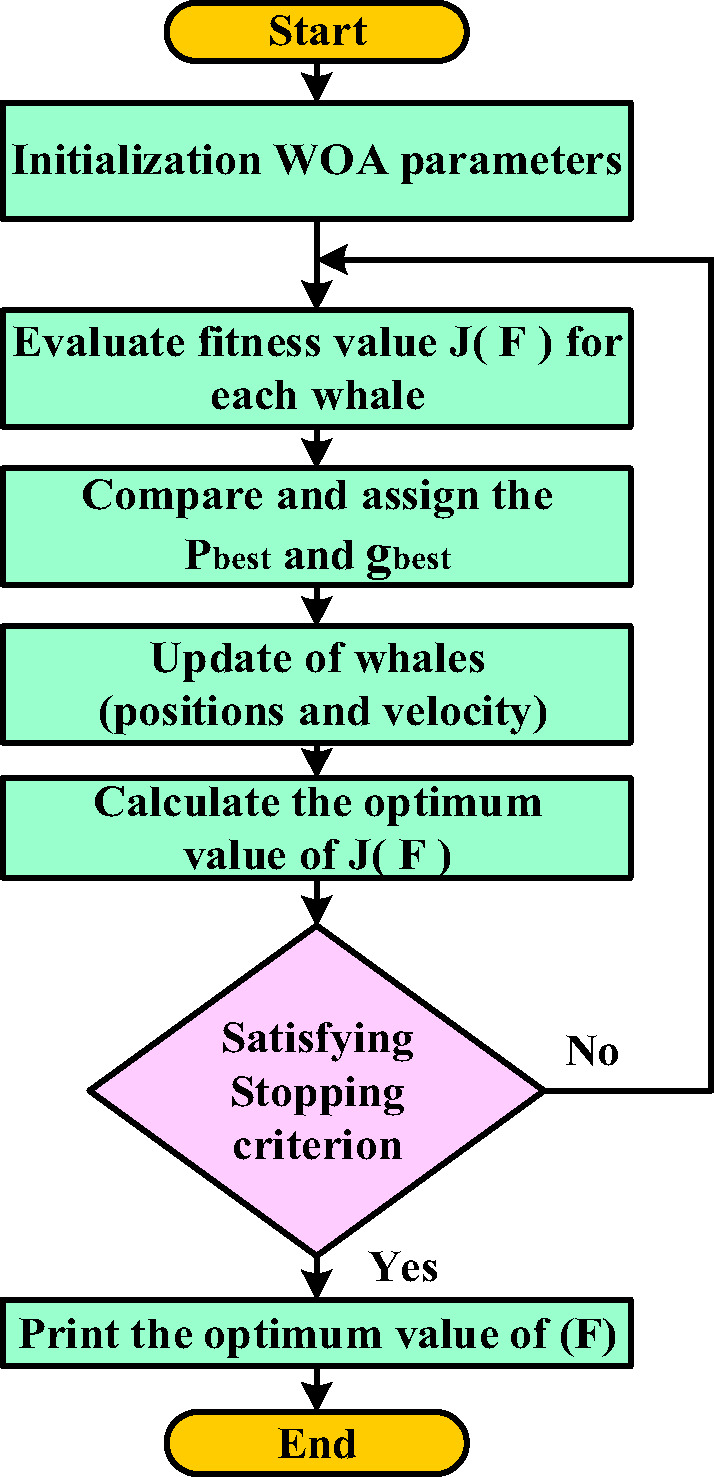
Flow chart of the WOA to calculate the best value of the scaling factor (F).

Since the scaling factor (*F*) significantly affects MPPT efficiency but does not have an exact analytical expression, a metaheuristic optimization method can be applied to find its best value. The WOA offers a reliable control mechanism across various load scenarios and system parameters. This enhances both the flexibility and resilience of the control framework, ensuring that the Beta MPPT method consistently performs at its best under changing operational conditions. The objective function aims to find the ideal value of the scaling factor in a way that enhances power extraction efficiency (*η*) while simultaneously reducing both convergence time (*CT*) and Steady-State Oscillations (*SSO*). The goal is to minimize *J(F)* and obtain the best value of the scaling factor as outlined in Fig. 7.

10 $${\text{J}}\left( {\text{F}} \right){\text{ }}= \times (1\, - \,\eta ){\text{ }}+ \times SSO$$

Where:

- MPPT Efficiency (*η*) is expressed as the ratio of the power obtained using the MPPT method ($\:{P}_{MPPT}$) to the ideal power ($\:{P}_{ideal}$).
- (*SSO*) is the Root Mean Square (RMS) value of the power fluctuations in the steady state.
- CT refers to the duration needed for the system to reach 98% of the $\:{\text{P}}_{\text{i}\text{d}\text{e}\text{a}\text{l}}$.
- (*W₁*,* W₂*, and *W₃* ) are the weighting coefficients assigned to balance the impact of each parameter in the optimization process.

#### Voltage source inverter

**Fig. 8:**
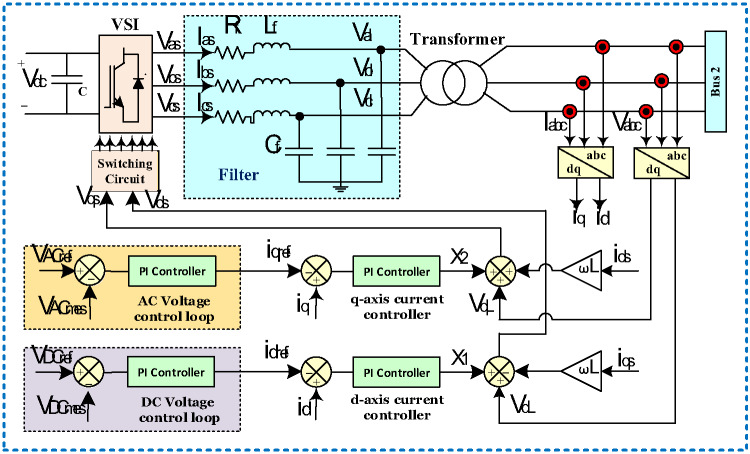
VSI control.

As illustrated in Fig. 8, the control framework of the voltage source inverter (VSI) includes two inner loops for managing current and two outer loops for managing voltage. The d-axis current ($\:{\text{I}}_{\text{d}}$) controls active power, which directly influences the DC bus voltage. On the other hand, controlling the q-axis current ($\:{\text{I}}_{\text{q}}$) allows for the regulation of reactive power, thus stabilizing the AC load voltage. The PI controller is employed to evaluate and enhance the dynamic response of the external voltage regulation loops on the DC and AC sides^3,21^. The mathematical expressions governing the VSI voltage are outlined in Eq. (11). To operate in the (dq) rotating reference frame (synchronous frame), the original three-phase (abc) signals are converted using transformation matrices, as described in Eq. (12).

11 $$\left[ {\begin{array}{*{20}{c}} {{V_{as}}} \\ {{V_{bs}}} \\ {{V_{cs}}} \end{array}} \right]=\left[ {\begin{array}{*{20}{c}} {{V_{aL}}} \\ {{V_{bL}}} \\ {{V_{cL}}} \end{array}} \right]{\text{+~}}{L_f}\frac{{{\text{~}}d}}{{dt{\text{~}}}}\left[ {\begin{array}{*{20}{c}} {{I_{as}}} \\ {{I_{bs}}} \\ {{I_{cs}}} \end{array}} \right]{\text{+~}}{R_f}\left[ {\begin{array}{*{20}{c}} {{I_{as}}} \\ {{I_{bs}}} \\ {{I_{cs}}} \end{array}} \right]$$

12 $$~\left[ {\begin{array}{*{20}{c}} {{V_{ds}}} \\ {{V_{qs}}} \end{array}} \right]=\left[ {\begin{array}{*{20}{c}} {{V_{dL}}} \\ {{V_{qL}}} \end{array}} \right]{\text{+~}}{L_f}\frac{{{\text{~}}d}}{{dt{\text{~}}}}\left[ {\begin{array}{*{20}{c}} {{I_{ds}}} \\ {{I_{qs}}} \end{array}} \right]{\text{~+~}}\omega {L_f}\left[ {\begin{array}{*{20}{c}} { - {I_{qs}}} \\ {{I_{ds}}} \end{array}} \right]{\text{+~}}{R_f}\left[ {\begin{array}{*{20}{c}} {{I_{ds}}} \\ {{I_{qs}}} \end{array}} \right]$$

Assume that $\:({V}_{as}$, $\:{V}_{bs}$, $\:{V}_{cs}$​) are the phase voltages produced by the VSI, and $\:{(I}_{as}$, $\:{I}_{bs}$, $\:{I}_{cs})$ ​ correspond to its output currents. The filter’s resistance and inductance are denoted by $\:{(R}_{f}$ and $\:{L}_{f})$ respectively. $\:{(V}_{aL}$, $\:{V}_{bL}$, $\:{V}_{cL})$​ are the voltages across the connected load. In the synchronous dq reference frame, $\:({V}_{dqs}$, $\:{V}_{dqL}$, $\:{I}_{dqs})$ ​ are the inverter’s output voltages, the load-side voltages, and the inverter output currents, respectively. According to the described approach, the control of reactive power is managed through the q-axis current component, as detailed in Eq. (13), while the regulation of active power is managed through the d-axis current, as specified in Eq. (14)^3,21^.

13 $${Q_s}=~ - \frac{3}{2}~\left[ {~{I_{qs}}{V_{ds}} - {I_{ds}}{V_{qs~}}} \right]$$

14 $${P_s}=~\frac{3}{2}~\left[ {~{I_{qs}}{V_{qs~}}+{I_{ds}}{V_{ds~}}} \right]$$

Here, $\:{(\text{Q}}_{s}$​ and $\:{\text{P}}_{s})\:$are the delivered reactive and active power, respectively. The responses generated by the current controllers aligned with the d-axis and q-axis are computed using the expressions provided in Eqs. (15) and (16)^3,21^.

15 $${X_1}=~{K_{p1}}~\left( {{I_d} - ~{I_{dref}}} \right)+{K_{i1}}\frac{z}{{z - 1}}~\left( {{I_d} - ~{I_{dref}}} \right)$$

16 $${X_2}=~{K_{p2}}~\left( {{I_d} - ~{I_{dref}}} \right)+{K_{i2}}\frac{z}{{z - 1}}~\left( {{I_d} - ~{I_{dref}}} \right)$$

### Modeling of the battery system

The integration of electrochemical storage units, such as lithium-ion battery banks, plays a pivotal role in HPS incorporating variable RES like PV arrays. These storage systems address imbalances between electricity production and consumption that arise from rapid fluctuations in solar insolation. During periods of diminished solar generation, when PV output falls short of the inverter’s target power level, the battery discharges to supplement load requirements^38,39^. Conversely, when PV generation exceeds demand, surplus energy is stored within the battery for next use. Solar installations inherently cease operation during nocturnal intervals due to the absence of sunlight^40,41^. Here, BATT synergizes with DEG to enhance system reliability and cost-effectiveness compared to standalone DEG configurations, reducing fuel consumption and operational expenses.

**Fig. 9:**
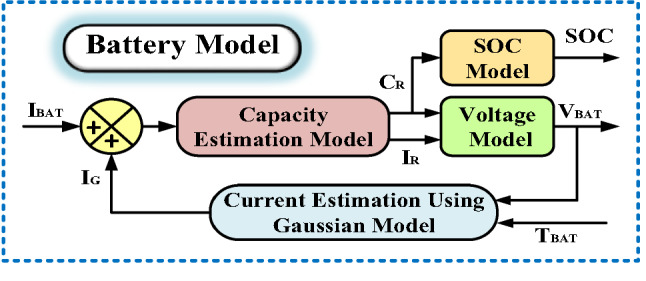
Battery Model.

The operational framework of the BATT, illustrated in Fig. 9, is governed by critical performance metrics including terminal voltage, energy capacity, and charge retention level State of Charge (SOC). The battery is mathematically represented as a tunable voltage source paired with an internal impedance component. Where $\:{(\text{C}}_{\text{R}}$ )​ is the rated capacity and $\:\left({\text{I}}_{\text{B}\text{A}\text{T}\text{T}}\right)$ ​is terminal current flow. Additional governing equations account for electrochemical reactions, gas evolution phenomena, thermal dynamics, and voltage-current relationships. Key variables include $\:{\text{V}}_{\text{B}\text{A}\text{T}\text{T}}$ ​ (battery terminal potential), $\:{\text{I}}_{\text{R}}$ ​ (internal reaction current), $\:{\text{I}}_{\text{G}}$ ​ (parasitic gassing current), and $\:{\text{T}}_{\text{B}\text{A}\text{T}\text{T}}$​ (operating temperature).

The battery management strategy enforces specific operational constraints to ensure safe and efficient usage. Firstly, it restricts both the charging and discharging power levels, ensuring they do not exceed the maximum threshold specified by Eq. (17). Secondly, as outlined in Eq. (18), it regulates the battery’s SOC, keeping it within acceptable boundaries to avoid risks associated with overcharging or excessive depletion^38–41^.

17 $${P_{bat.min}} \leqslant {P_{bat}}\left( t \right){\text{~}} \leqslant {\text{~}}{P_{bat.max}}$$

18 $$SO{C_{min}} \leqslant SOC\left( t \right){\text{~}} \leqslant {\text{~}}SO{C_{max}}$$

**Fig. 10:**
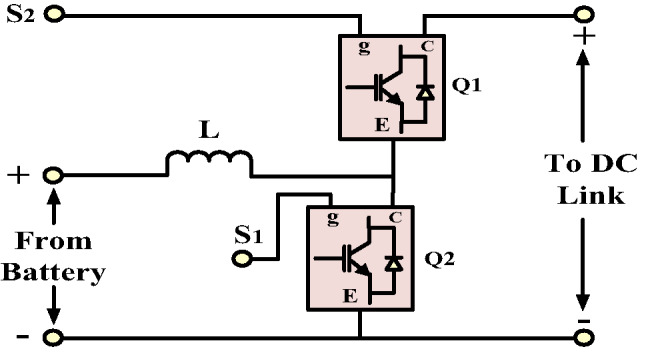
Bi-directional DC-DC converter.

**Fig. 11:**
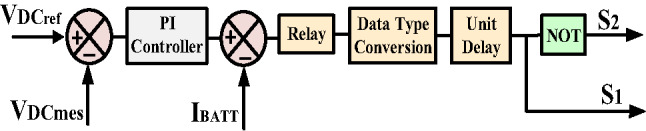
Battery controller.

In the proposed system, batteries are utilized to mitigate the effects of the intermittent nature associated with PV sources. Due to their high energy density, batteries can deliver power at nearly constant voltage when their charging and discharging cycles are appropriately managed. The modeled battery is integrated into the DC link through a bi-directional DC-DC converter, as illustrated in Fig. 10. This converter facilitates the charging and discharging of the battery while maintaining the DC link voltage at 500 volts. When the battery supplies power to the microgrid, the converter operates in boost mode; conversely, when it absorbs power from the grid or PV panels, it operates in buck mode. The control loop regulates the DC link voltage by adjusting the duty cycle of the bi-directional DC-DC converter. It continuously measures the DC link voltage, compares it to a reference value, and processes the error through a voltage mode compensator to determine the necessary duty ratio. This control approach is agnostic to the direction of power flow and generates appropriate switching signals for the buck and boost operations. As shown in Fig. 11, an intelligent controller determines the operational mode and transmits the control pulses to a designated semiconductor switch. The decision to operate the converter in a buck or boost mode is based on the command signal received from the HPS. In the absence of a regulation signal, the battery’s SOC determines whether the converter should operate in buck mode to facilitate charging.

### Modeling of the DEG system

The DEG assumes a crucial role as a backup power solution, particularly in scenarios where RES such as PV is insufficient due to intermittent availability or environmental factors. Additionally, the system activates in island mode when the main grid experiences instability, such as voltage sags, frequency deviations, or unforeseen disconnections. In this isolated operational state, the DEG autonomously sustains power supply to critical loads, preventing blackouts and enabling seamless transitions until grid conditions stabilize or renewable generation resumes. This dual functionality underscores the DEG’s importance in hybrid energy systems, bridging gaps between renewable intermittency and grid reliability while ensuring uninterrupted electricity access during emergencies^42,43^.

**Fig. 12:**
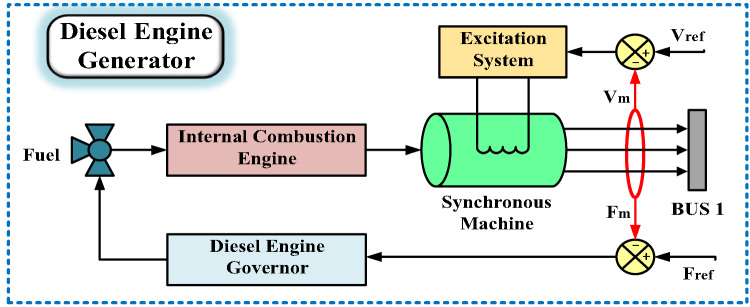
Diesel Engine Generator model.

The DEG system illustrated in Fig. 12 is composed of multiple interconnected elements designed to ensure reliable power generation and grid stability. At its core, the system includes a governor mechanism for the diesel engine, an excitation system, and a synchronous machine integrated with the engine. The governor operates through a closed-loop feedback control strategy, which continuously monitors and adjusts the engine’s rotational speed. By dynamically aligning the engine’s output with a predefined reference speed, the governor guarantees the stabilization of the electrical grid’s frequency, even under fluctuating load demands. This precision in speed regulation is critical for maintaining synchronization between the generator and the grid, thereby preventing disruptions in power quality.

## Frequency control

The primary objective of stabilizing an islanded AC HPS lies in regulating the electrical supply to preserve system frequency at its predefined operational standard. This process hinges on frequency stability management, which entails dynamically modulating generator output levels to equilibrate power consumption needs while sustaining consistent grid oscillations. Within such systems, the cumulative energy contribution from distributed resources—comprising DEG, PV, and BATT—must collectively satisfy load requirements, as expressed by the relationship:

19 $${P_{Load}}=~{P_{DEG}}+~{P_{PV}} \pm {P_{BATT}}$$

Given the inherent variability of PV generation due to weather-dependent intermittency, this analysis prioritizes DEG as the primary actuator for frequency correction. The control framework compensates for deviations caused by fluctuating loads and PV generation by adaptively scaling DEG output. Conventional PI regulators remain widely adopted for such stabilization tasks, while FPI systems introduce rule-based adaptability, enhancing responsiveness to dynamic operational shifts. To address limitations in existing hybrid energy systems, this work proposes an MRAC-FPI-WOA framework, which synergizes adaptive reference tracking with fuzzy logic to optimize disturbance rejection across diverse instability scenarios.

PI-PSO, PI-WOA, and FPI-WOA architectures have proved efficacy in grid frequency management, yet the MRAC-FPI-WOA hybrid appears as a superior solution, using real-time parameter adaptation to maintain precision under abrupt load transitions, resource volatility, and compound disruptions. This innovation underscores the critical need for advanced control paradigms in modernizing HPS resilience against the uncertainties of renewable integration.

### Conventional PI controller

The study specifically examines the PI controller’s effectiveness in maintaining system frequency stability and enhancing proposed IHPS operational performance, utilizing a control law expressed as (Eq. 20), with particular focus on its PI controller dynamic response characteristics and stabilization capabilities under varying load conditions^4^.

20 $$u\left( t \right)={k_p}~e\left( t \right)+\mathop \smallint \limits_{0}^{t} {k_i}~e\left( t \right)dt$$

This equilibrium enables accelerated convergence and superior precision compared to conventional optimization frameworks. By defining frequency control as an optimization problem, the ITAE performance metric can be minimized^44,45^.

21 $$ITAE={\text{~}}\mathop \smallint \limits_{0}^{T} t{\text{~}}\left| {e\left( t \right)} \right|d\left( t \right)$$

Where (t) is time, while e(t) is the deviation between $\:{F}_{m}\:$and $\:{F}_{ref}$.

#### PI-PSO controller

**Fig. 13:**
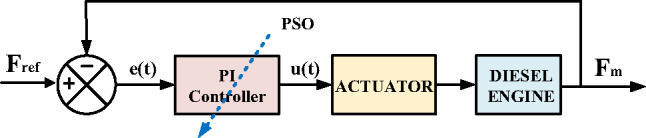
PI-PSO Controller-Based Control System Structure.

The system configuration depicted in Fig. 13 presents the closed-loop control structure employing the PI-PSO controller. PSO is popular for its simplicity and fitness-based approach, effective for diverse optimization problems. However, it risks premature convergence due to declining swarm diversity. The methodology incorporates three fundamental components^46^:

- Individual Best ($\:{\:P}_{pest\:}$): The optimal solution encountered by particle (i) during its search history.
- Global Best ($\:{\:g}_{pest\:}$): The most favorable solution discovered by the entire particle collective.
- Dynamical Update Rules: Governing equations directing particle movement through the solution space.

The particle’s velocity vector is modified following Eq. (22), while its positional coordinates are recomputed via Eq. (23) through vectorial addition of the updated velocity to its prior location^47^.

22 $$~{v_{id}}\left( {1+t} \right)=~w{v_{id}}\left( t \right)+~{r_1}{C_1}\left( {~{P_{pest,id}}\left( t \right) - ~~{X_{iid}}\left( t \right){\text{~}}} \right)+{\text{~}}~{r_2}{C_2}\left( {~{g_{pest,id}}\left( t \right) - ~~{X_{id}}\left( t \right){\text{~}}} \right){\text{~~~~}}~~~~d=1,2, \ldots .D~{\text{~}}$$

23 $$~{X_{id}}\left( {1+t} \right)=~{X_{id}}\left( t \right)+~{v_{id}}\left( {1+t} \right){\text{~~~~~~~~~~~~~~~~}}~d=1,2, \ldots .D~$$

**Fig. 14:**
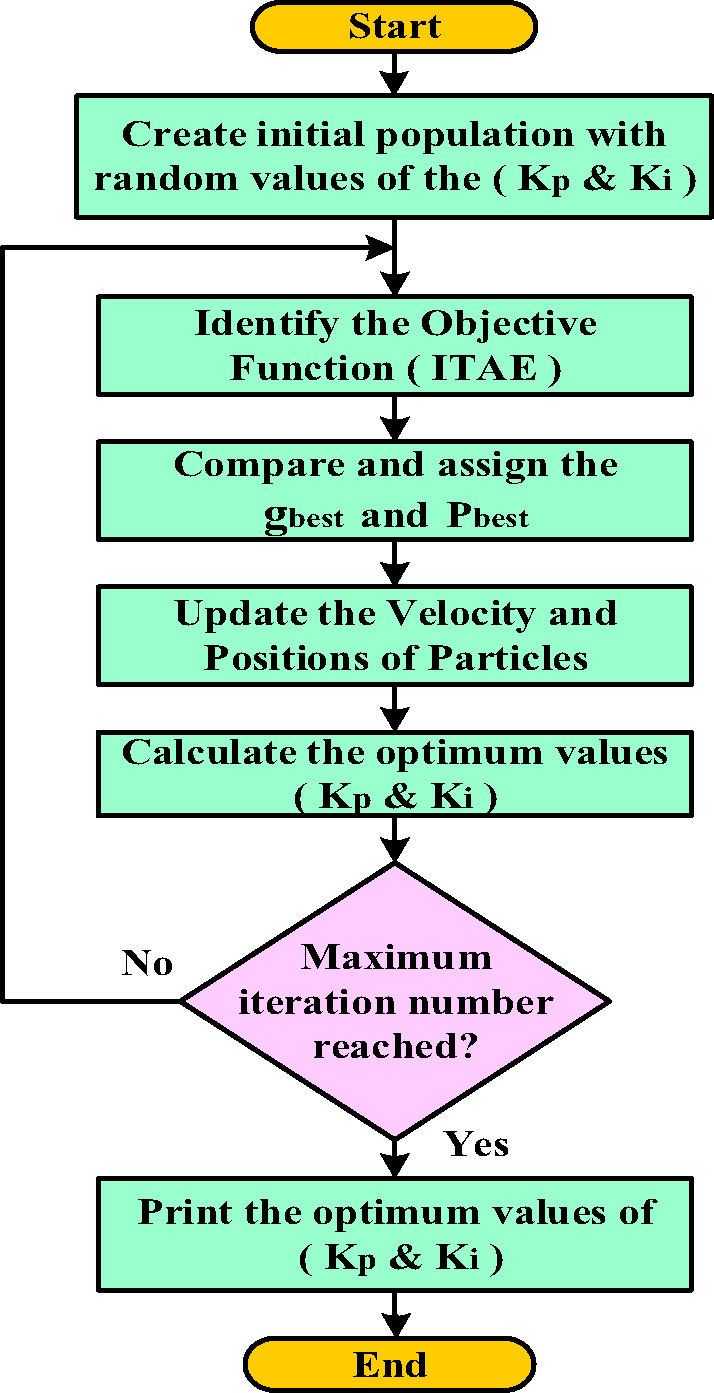
PSO flowchart.

The PSO algorithm updates each particle’s velocity and position through three key components: (1) an inertia term ($\:{\:wv}_{id}$) that preserves momentum from previous movements, (2) a cognitive component ($\:{\:r}_{1}{C}_{1}\left({\:P}_{pest,id}\left(t\right)-\:{\:X}_{iid}\left(t\right)\:\right)$)) that attracts particles toward their personal best positions ($\:{P}_{pest,id}$), and (3) a social component ($\:{\:r}_{2}{C}_{2}\left({\:g}_{pest,id}\left(t\right)-\:{\:X}_{id}\left(t\right)\:\right)$)) that guides particles toward the swarm’s global best solution ($\:{g}_{pest,id}$), where (w) represents the inertia weight, C₁ and C₂ are cognitive and social learning rates, respectively, and (r_1_,r_2_) are random numbers that maintain stochastic exploration. This balanced combination of individual experience (cognitive) and collective knowledge (social) enables effective search-space exploration while progressively converging toward optimal solutions. Figure 14 illustrates the algorithm’s operational flowchart^48^.

#### PI-WOA controller

**Fig. 15:**
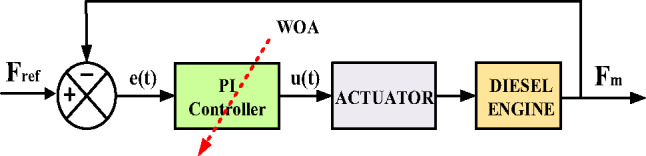
PI-WOA Controller-Based Control System Structure.

PI-WOA controller illustrated in Fig. 15, for frequency stabilization. By framing the controller tuning process as an optimization problem, WOA dynamically minimizes frequency deviations through iterative adjustments to the gain values, ensuring robust adaptability to grid disturbances. This hybrid approach synergizes the simplicity of PI control with the intelligence of bio-inspired optimization, enabling enhanced precision in frequency regulation for modern power networks characterized by intermittent renewable integration and complex load dynamics. The methodology aims to elevate grid resilience, reduce oscillations, and maintain nominal frequency stability under heterogeneous operating conditions.

WOA technique is a robust nature-inspired computational method modeled after the foraging strategies of humpback whales. It shows exceptional ability in addressing intricate optimization problems by harmonizing the search for novel solutions (exploration) with the refinement of existing ones (exploitation)^49,50^.

**Fig. 16:**
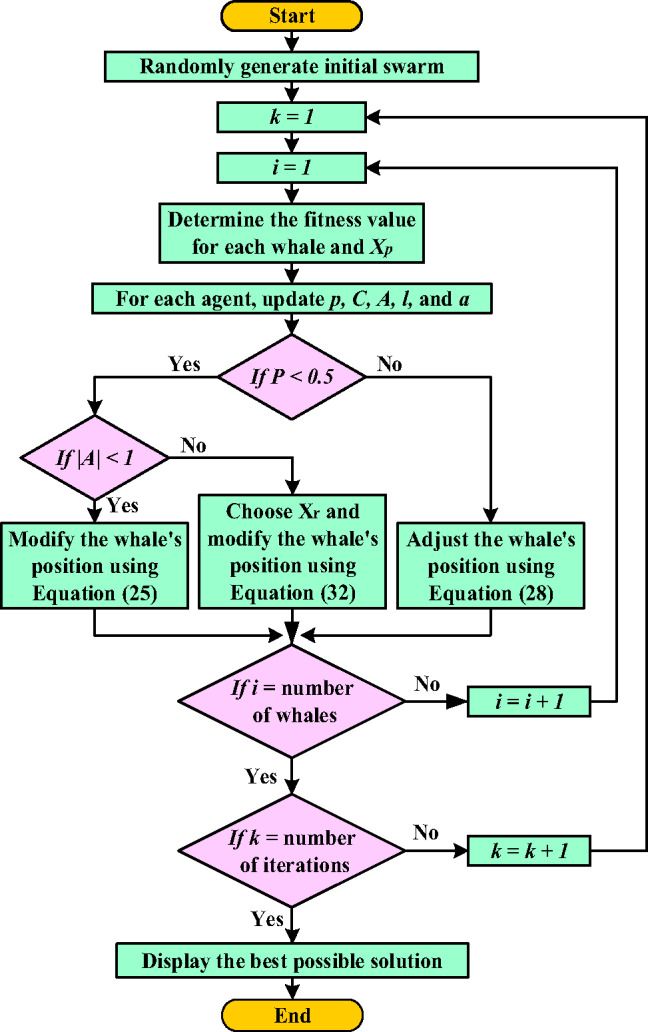
WOA flow chart.

Figure 16 illustrates the flowchart of the WOA, which can be mathematically expressed using the following Eqs^49,50^.

24 $$D=\left| {C{\text{~}}.{\text{~}}{X_P}\left( t \right) - X\left( t \right)} \right|$$

25 $$X\left( {t+1} \right)={\text{~}}{X_P}\left( t \right) - D.A$$

26 $$A=2a~.r~ - a$$

27 $$C=2~.r$$

28 $$X\left( {t+1} \right)=~{X_P}\left( t \right)+D^{\prime}.~{e^{bl}}.\cos \left( {2\pi l} \right)$$

29 $$D^{\prime}=~\left| {{X_P}\left( t \right) - ~X\left( t \right)} \right|$$

30 $$X\left( {t+1} \right)~\left\{ {\begin{array}{*{20}{c}} {D^{\prime}.~{e^{bl}}.\cos \left( {2\pi l} \right)+{X_P}\left( t \right)~~~if~p~ \geqslant o.5~} \\ {{X_P}\left( t \right) - A~.D~~~~~~~~~~~~~~~~~~~~~~if~p~<o.5} \end{array}~} \right.$$

31 $$D=\left| {C~{X_r}\left( t \right) - X\left( t \right)~} \right|$$

32 $$X\left( {t+1} \right)=~{X_r}\left( t \right) - A~.D~$$

**Fig. 17:**
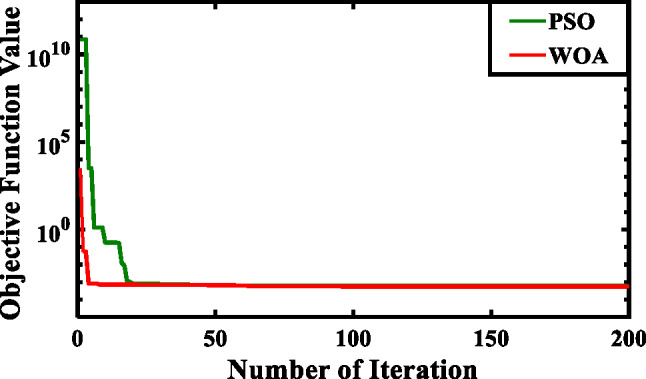
Convergence of the objective function.

The symbols X(t), $\:{X}_{p}\left(t\right)$, and $\:{X}_{r}\left(t\right)$, correspond to the position vectors of the whale, prey, and random whale, respectively. (t) is the current iteration. (A and C) are the coefficient vectors. Over the number of rounds, (a) constantly decreases linearly from 2 to 0. The random integer (l) is between − 1 and 1, the random vector (r) is between 0 and 1, the (p) is the probability number ε [0, 1], and the constant that determines the spiral logarithmic form is represented by (b). Figure 17 demonstrates the convergence behavior of the objective function for both optimization methods, with Table 1 detailing the corresponding algorithmic parameters and optimized PI controller gains obtained through WOA and PSO implementations.

**Table 1:** The parameters of PSO and WOA.

PSO		WOA	
Parameter	Value	Parameter	Value
Population Size	50 particles	Population Size	50 whales
Maximum Iterations	200	Maximum Iterations	200
Inertia weight upper bound	0.9	Coefficient Range (a)	2 ˃ a ˃ 0
Inertia weight Lower bound	0.4	Random Integer (l)	1 ˃ I ˃ -1
Cognitive coefficient (C1)	1.5	Random Number (r)	1 ˃ r ˃ 0
Social coefficient (C2)	1.5	P-value	0.5
Upper bound	[2000, 2000]	Upper bound	[2000, 2000]
Lower bound	[0, 0]	Lower bound	[0, 0]

### FPI-WOA controller

**Fig. 18:**
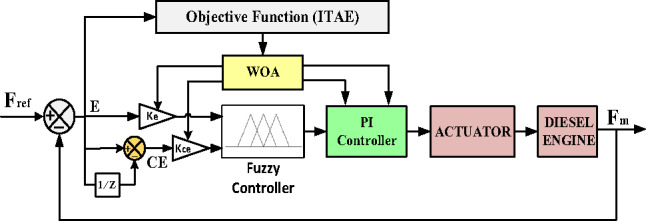
FPI-WOA Controller-Based Control System Structure.

**Fig. 19:**
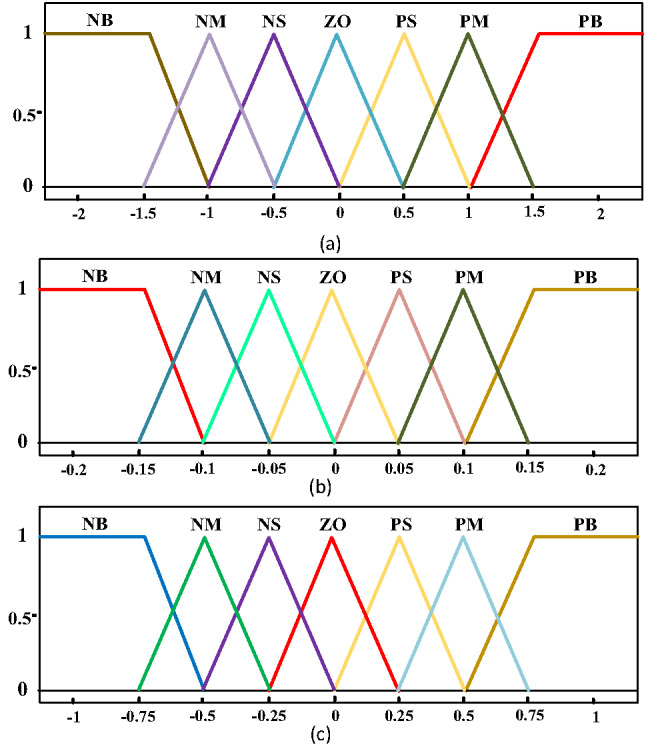
The MFs (**a**) E, (**b**) CE, (**c**) ΔD.

This research explores the FPI-WOA controller, illustrated in Fig. 18, as a hybrid control strategy that merges essential aspects of both FLC and PI-WOA control frameworks, aiming to enhance the capabilities of the PI controller by incorporating the advantages of FPI control. FLC outperforms classical methods in complex power systems due to its adaptability to nonlinearities and uncertainties without precise modeling. They maintain robust performance amid variable conditions like renewable generation fluctuations and load changes. Their rule-based heuristic approach enables intuitive tuning using operational expertise rather than complex math. Additionally, they are less sensitive to parameter variations than PID controllers, making them ideal for real-world applications with drifting system parameters^2,20,21^. As outlined in^51,52^ the fuzzy inference process consists of three main phases. The first step, fuzzification, transforms precise input values into fuzzy variables within their respective fuzzy sets. In this study, two input Errors (E), depicted in Fig. 19(a), and a Change in Error (CE), illustrated in Fig. 19(b), along with one output, shown in Fig. 19(c), are represented through triangular membership functions. Each input and output is characterized through a set of seven linguistic levels: NB (Negative Big), NM (Negative Medium), PB (Positive Big), PM (Positive Medium), PS (Positive Small), NS (Negative Small), and ZO (Zero). At the fuzzy logic rule inference stage, decisions are formulated through the integration of aggregation and implication techniques within the framework of fuzzy inference rules. The fuzzy rules, detailed in Table 2, can be linguistically described as follows: If both error (E) and (CE) are categorized as (PB), then the corresponding output is also classified as (PB). The parameters of PI-PSO, PI-WOA, and FPI-WOA controllers are displayed in Table 3.

**Table 2:** Rule matrix for the developed FLC.

Error	Change of Error						
	NB	NM	NS	ZO	PS	PM	PB
NB	NB	NB	NB	NB	NM	NS	ZO
NM	NB	NB	NB	NM	NS	ZO	PS
NS	NB	NB	NM	NS	ZO	PS	PM
ZO	NB	NM	NS	ZO	PS	PM	PB
PS	NM	NS	ZO	PS	PM	PB	PB
PM	NS	ZO	PS	PM	PB	PB	PB
PB	ZO	PS	PM	PB	PB	PB	PB

**Table 3:** Controller parameters resulting from PSO and WOA.

Controller type	$\:\text{P}\text{a}\text{r}\text{a}\text{m}\text{e}\text{t}\text{e}\text{r}\text{s}$			
	Kp	Ki	Ke	Kce
PI-PSO	59.184	153.641	-------	-------
PI-WOA	18.382	214.973	-------	-------
FPI-WOA	13.798	427.866	1.837	0.652

### MRAC-FPI-WOA controller

To enhance the accuracy of the FPI-WOA controller, it has been integrated with MRAC to enhance its efficiency and adaptability. Implementing MRAC in IHPS offers significant benefits, particularly in managing the unpredictable nature of RES. By dynamically adjusting to changes in generation and load variations, MRAC strengthens frequency stability and voltage regulation, optimizing system performance through continuous tuning of control variables adjusted during real-time operating conditions. This integration results in a more robust and dependable energy system, enabling seamless RES integration while improving the overall efficiency and reliability of IHPS.

**Fig. 20:**
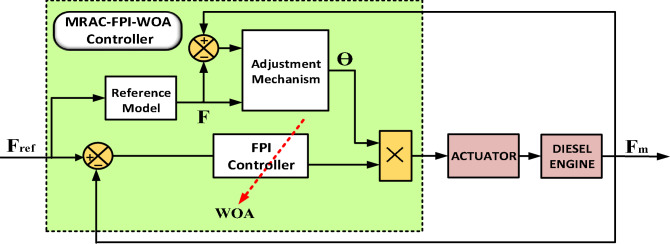
MRAC-FPI-WOA Controller-Based Control System Structure.

Significant applications include maintaining stable output voltage in DC-DC converters used in IHPS^53^, implementing a tailored MIT-rule-driven MRAC for boost-type DC-DC converters^54^, and improving conventional droop-based regulation in marine power systems^55^. Additionally, MRAC has been applied in HPS to regulate the unified interphase power controller (UIPC)^56^, and develop a fractional-order MRAC control strategy to stabilize voltage and current in multi-source power configurations using DC-DC converters^57^. As depicted in Fig. 20, the MRAC-FPI-WOA controller consists of three main components: the FPI controller, the reference model, and the adjustment mechanism.

## Results and discussion

**Fig. 21:**
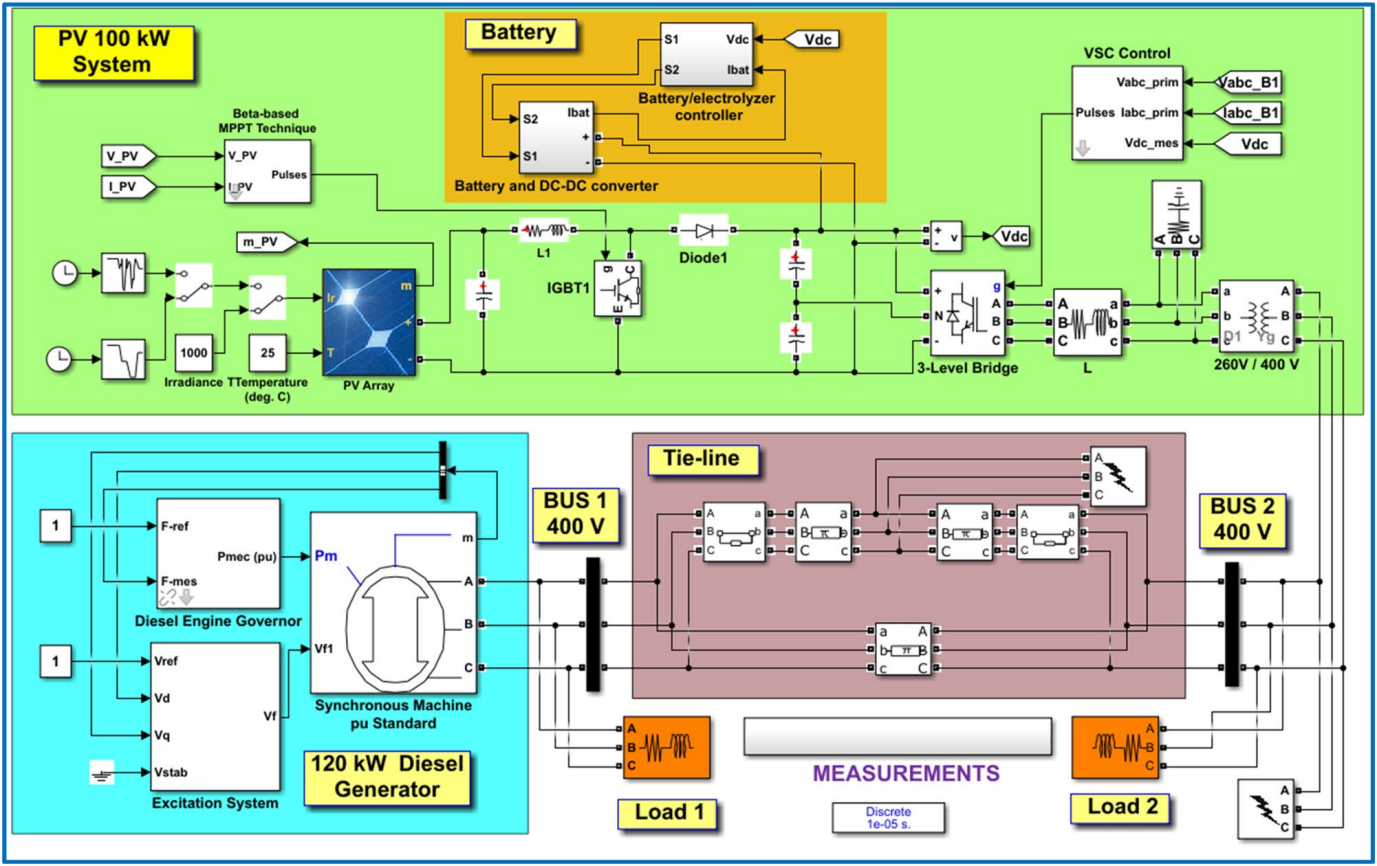
MATLAB/Simulink model.

In this study, simulations were conducted using MATLAB Simulink as shown in Fig. 21 to introduce an MRAC-FPI-WOA controller designed to ensure frequency stability within the system while facilitating fundamental control processes. A comparative analysis was performed between the MRAC-FPI-WOA, FPI-WOA, PI-WOA, and PI-PSO controllers across various scenarios to analyze the controllers’ effectiveness. These scenarios are essential for a thorough assessment of performance. For example, Case 1, which focuses on a three-phase fault at Bus 2, offers insights into the system’s robustness across different network configurations. Case 2 analyzes a three-phase fault occurring at the center of the tie-line, further evaluating the system’s capacity to manage faults that impact multiple components at once. Additionally, Cases 3, 4, and 5 address fluctuations in solar radiation, including step changes, ramp changes, and random variations, respectively. These scenarios are crucial for understanding how dynamic solar input influences overall system performance, given the inherent variability of solar energy due to environmental factors. Case 6 introduces a rapid load change, testing the system’s responsiveness to sudden alterations in energy demand, a frequent challenge in practical applications. Finally, Case 7 merges Cases 3 and 6, running them simultaneously to evaluate how the system performs under various conditions of concurrent changes in solar radiation and load demands. This integrated approach offers a comprehensive understanding of how various disturbances interact and impact frequency regulation, ultimately informing more efficient design and control strategies for the proposed IHPS. This section presents an in-depth analysis that includes a variety of responses and numerical results, demonstrating the findings and implications of our research. The nominal specifications for the PV, DEG, BATT, and loads are detailed in Table 4.

**Table 4:** IHPS specifications were used in the simulation.

Category		Parameters		Value	Parameters		Value
PV	PV Module	SunPower SPR-305E-WHE-D Module					
		Series-connected modules in a string		5	Voltage at MPP ($\:{\text{V}}_{\text{M}\text{P}\text{P}}$) (V)		54.7
		Parallel strings		66	Current at MPP ($\:{\text{I}}_{\text{M}\text{P}\text{P}}$) (A)		5.58
		Maximum Power (W)		305.266	Short-circuit current ($\:{\text{I}}_{\text{s}\text{c}}$) (A)		5.96
		Series resistance ($\:{\text{R}}_{\text{s}\text{e}\text{r}}$) (Ω)		0.37152	Open circuit voltage ($\:{\text{V}}_{\text{o}\text{c}}$) (V)		64.2
		Diode ideality factor		0.94504	Shunt resistance ($\:{\text{R}}_{\text{s}\text{h}}$) (Ω)		269.5934
	Boost Converter	Switching frequency (kHz)		5	resistance (Ω)		0.005
		Capacitance (µF)		100	Inductance (mH)		5
	Inverter	Switching frequency (kHz)		10	Number of bridge arms		3
		Snubber resistance (MΩ)		1	Snubber capacitance $\:{\text{C}}_{\text{s}}$ (F)		infinity
		Power Electronic device		IGPT	Internal resistance (mΩ)		0.2
	Transformer	Nominal power (kVA)		100	Nominal frequency (Hz)		50
		V1 Ph-Ph ($\:{\text{V}}_{\text{r}\text{m}\text{s}}$)		260	V2 Ph-Ph ($\:{\text{V}}_{\text{r}\text{m}\text{s}}$)		400
DEG	Synchronous Machine	Rated power ($\:{\text{P}}_{\text{D}\text{E}\text{G}}$)		120 kW	Frequency rating ($\:{\text{F}}_{\text{D}\text{E}\text{G}}$)		50 Hz
		Rated voltage ($\:{\text{V}}_{\text{D}\text{E}\text{G}}$)		400 V	Pole pairs (P)		1
		Friction factor		0	Stator resistance (pu)		0.003
	Excitation System	Low-pass filter time constant $\:{\text{T}}_{\text{r}}$(ms)		20	Exciter	$\:{\text{K}}_{\text{e}}$	1
						$\:{\text{T}}_{\text{e}}$(s)	0
		Regulator gain	$\:{\text{K}}_{\text{a}}$	300	Transient gain reduction	$\:{\text{T}}_{\text{b}}$(s)	0
		Regulator time constant	$\:{\text{T}}_{\text{a}}$(s)	0.001		$\:{\text{T}}_{\text{c}}$(s)	0
		Damping filter gain ($\:{\text{K}}_{\text{f}}$)		0.001	Damping filter time constant ($\:{\text{T}}_{\text{f}}$)		0.1
		Terminal Voltage Initial Value		1	Field Voltage Initial Value		1.2473
	Diesel Engine Governor	Actuator time constants	T4	0.25	initial value of mechanical power Pm0 (pu)		0.1361
					Torque limits	T_min_ (pu)	0
			T5	0.009		T_max_ (pu)	1.1
			T6	0.0384	Engine time delay T_d_ (s)		0.024
BATT		Rated Capacity (PC) (Ah)		50	Initial state-of-charge (%)		60%
		Rated voltage ($\:{\text{V}}_{\text{B}\text{A}\text{T}\text{T}}$) (V)		300	Battery response time (s)		30
		Discharge					
		Maximum capacity (Ah)		53.8462	Rated Discharge Current (A)		10
		Minimum Operating Voltage (V)		225	Internal Resistance (Ω)		0.06
		Peak charged voltage (V)		353.389	Capacity (Ah) at nominal voltage		48.0769
Loads		Load1		50 kW	Load 2		70 kW
		Power Factor		0.9	Power Factor		0.9
HPS		Microgrid Frequency ($\:{\text{F}}_{\text{H}\text{P}\text{S}}$)		50 Hz	Microgrid voltage ($\:{\text{V}}_{\text{B}\text{U}\text{S}}$)		400 V

### Case 1. Three-phase fault on BUS 2

**Fig. 22:**
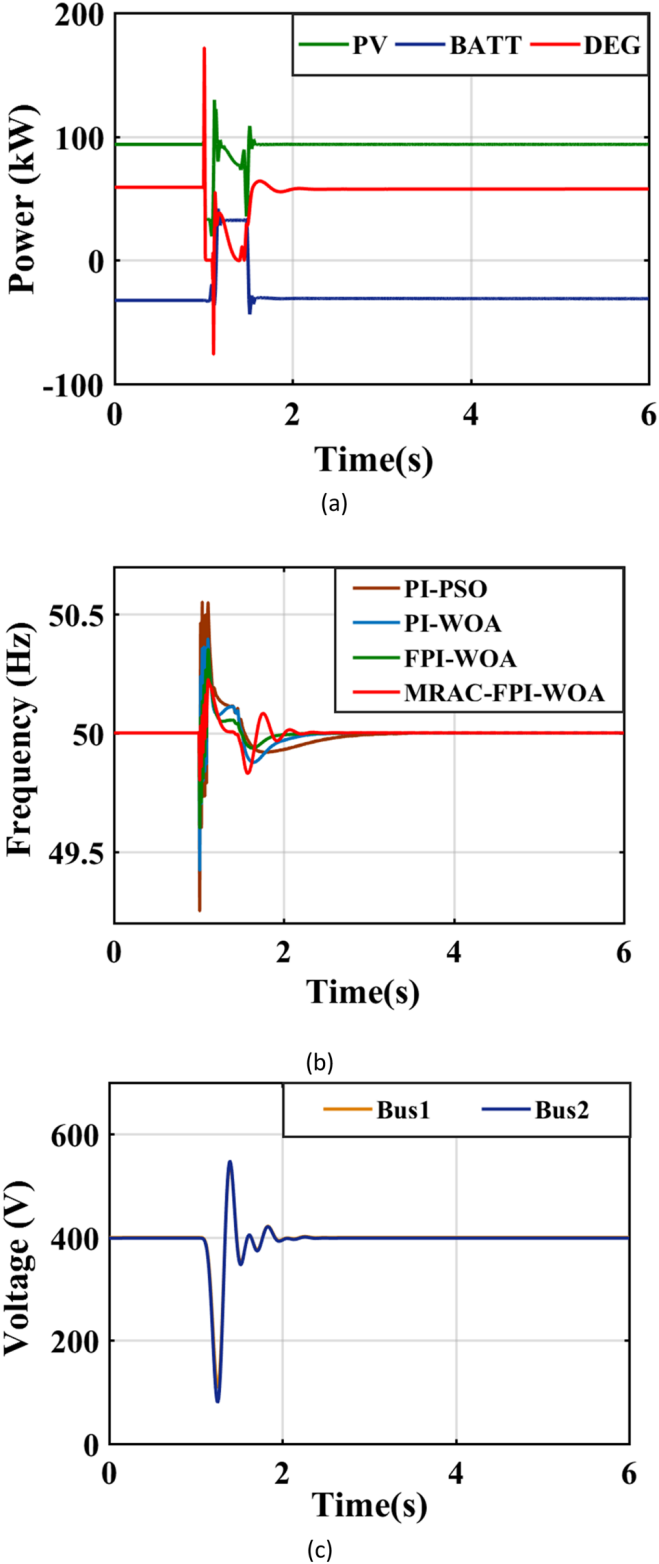
System behavior in case 1. (**a**) Output power of the sources, (**b**) System Frequency, (**c**) System Voltage.

In this situation, a fault involving a three-phase short circuit at BUS 2 occurs, lasting 0.1 s. This fault starts at the terminals of the AC load after a time interval of 1 s and is identified by a fault resistance of 0.001 ohms. Figure 22(a) visually illustrates the output power generated by both the PV and DEG sources. Additionally, Table 5; Fig. 22(b) assess the overall efficiency of the system’s frequency by analyzing various control strategies, including the MRAC-FPI-WOA, FPI-WOA, PI-WOA and PI-PSO controllers. This evaluation considers several Key performance indicators like ITAE, $\:{\text{T}}_{\text{s}}$, %$\:{\text{M}}_{\text{p}}$, and %$\:{\text{M}}_{\text{u}\text{s}}\:$during instances of three-phase faults. The findings from this case prove that the MRAC-FPI-WOA controller surpasses the PI-WOA, PI-PSO and FPI-WOA controllers in every evaluated aspect. Furthermore, Fig. 22(c) shows the voltage values existing in the system.

### Case 2. A three-phase fault happens in the center of the tie line

**Fig. 23:**
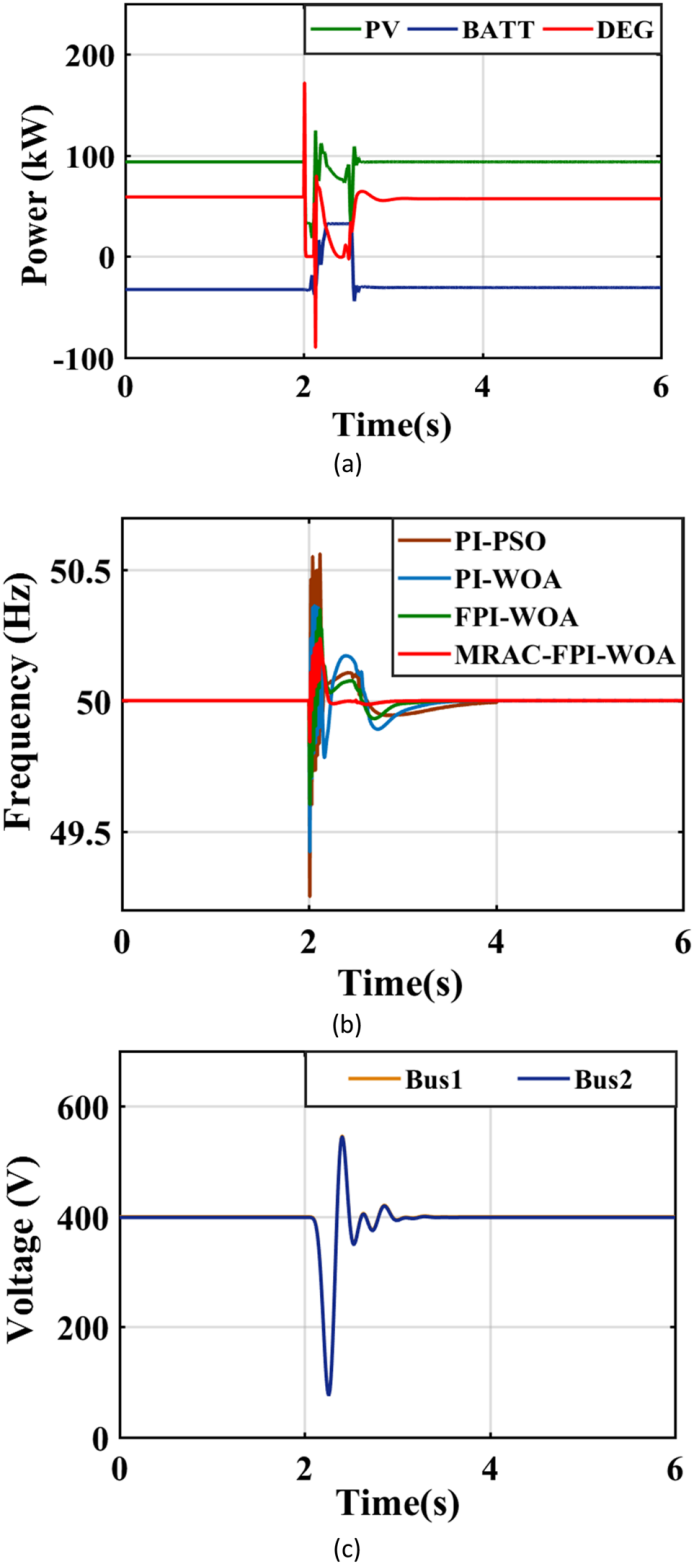
System behavior in case 2. (**a**) Output power of the sources, (**b**) System Frequency, (**c**) System Voltage.

In this case, a three-phase short circuit starts at the central point of the tie, lasting for a total duration of 0.11 s. This fault event starts at the terminals of the AC load after 2 s and shows fault resistance as low as 0.001 ohms. Figure 23(a) visually depicts the output power generated by both the PV and DEG sources. Meanwhile, Table 5; Fig. 23(b) provide an assessment of the system’s efficiency by examining various control methodologies, including the MRAC-FPI-WOA, FPI-WOA, PI-WOA and PI-PSO controllers. The evaluation process considers several important performance metrics, such as ITAE, %$\:{\text{M}}_{\text{u}\text{s}}$, %$\:{\text{M}}_{\text{p}}$, and $\:{\text{T}}_{\text{s}}$, specifically during instances of three-phase faults. The results from this analysis confirm the enhanced effectiveness of the MRAC-FPI-WOA controller over other controllers in all evaluated performance aspects. Additionally, Fig. 23(c) illustrates the voltage values existing in the system.

### Case 3. Step changes in solar radiation

**Fig. 24:**
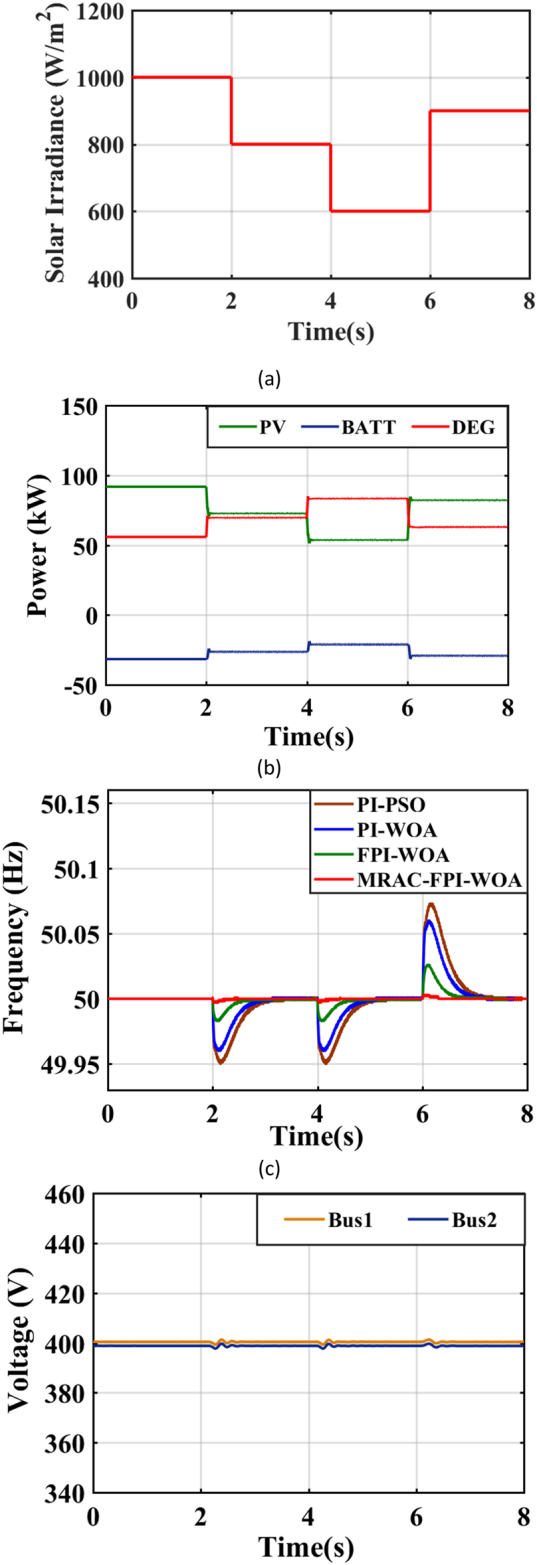
System behavior in case 3. (**a**) Radiation is a step-changed profile, (**b**) Output power of the sources, (**c**) System Frequency, (**d**) System Voltage.

Figure 24(a) illustrates the stepped variation in solar irradiance over time. One must note that changes in solar radiation levels can significantly affect the frequency within the system. The implementation of the MRAC-FPI-WOA controller plays a vital role in ensuring effective frequency regulation under these varying conditions. When compared to PI-PSO, PI-WOA and FPI-WOA controllers, the MRAC-FPI-WOA controller demonstrates a higher level of accuracy in responding to abrupt changes in solar radiation, notably when efficiency is high and weather conditions shift rapidly. The performance efficiency is assessed using the control strategies, taking into account several key parameters, including %$\:{\text{M}}_{\text{u}\text{s}}$, ITAE, $\:{\text{T}}_{\text{s}}$, %$\:{\text{M}}_{\text{p}}$. Table 5; Fig. 24(b) present an in-depth analysis of the IHPS frequency’s behavior in response to a step change. Furthermore, Fig. 24(c) visually represents the output power produced by both the PV and DEG sources. In this scenario, when solar radiation diminishes from 1000 to 800 W/m² after two seconds, the PV power decreases from 94 kW to 73 kW. This reduction in PV power generation prompts an increase in the power output from the DEG, which grows from 56 kW to roughly 70 kW to meet the energy demand. Conversely, when solar radiation declines further to 600 W/m² after an additional four seconds, while maintaining a constant ambient temperature, the PV power output declines from 73 kW to about 54 kW. In response, the DEG power generation escalates from 70 kW to approximately 84 kW to satisfy the demand. Additionally, when solar radiation increases from 600 to 900 W/m² at the six-second mark, PV power rises from 54 kW to about 82 kW, causing the DEG output to decline from 84 kW to roughly 63 kW. The proposed controller successfully stabilizes the system frequency, even amidst fluctuations in solar radiation levels. Lastly, Fig. 24(d) provides further insight by presenting the distribution of voltage across the system.

### Case 4. Ramp changes in solar radiation

**Fig. 25:**
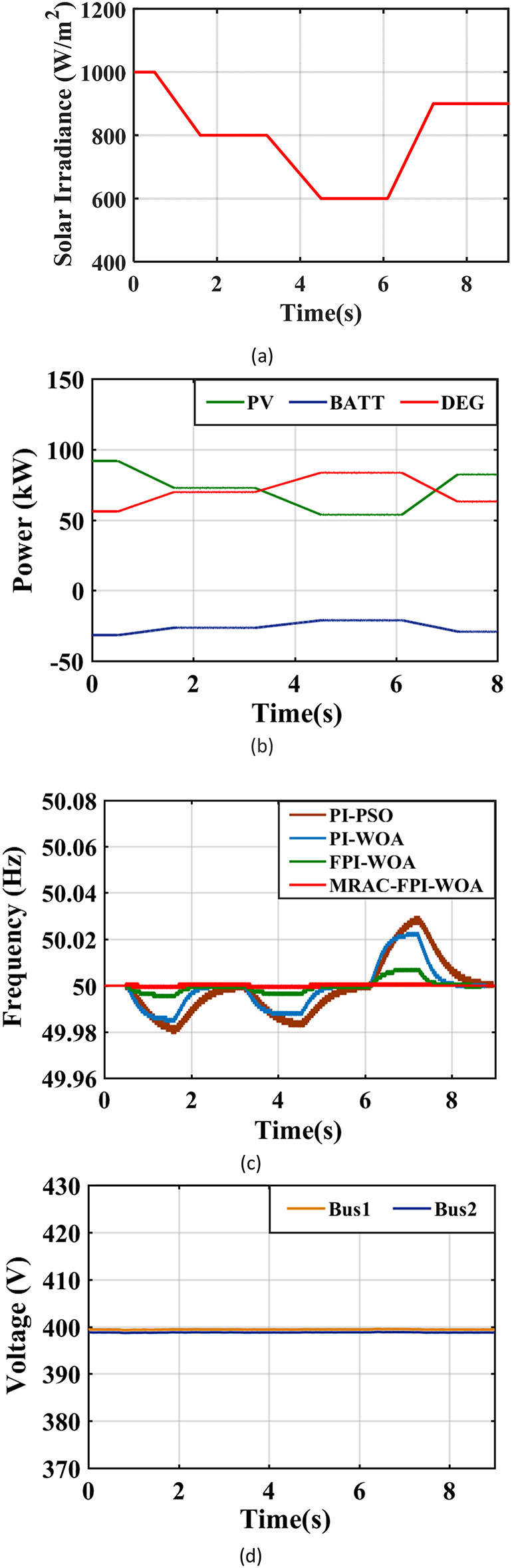
System behavior in case 4. (**a**) Radiation is a ramp-changed profile, (**b**) Output power of the sources, (**c**) System Frequency, (**d**) System Voltage.

Figure 25(a) illustrates the ramp-shaped trend of solar irradiance over a specific period. The MRAC-FPI-WOA controller plays a crucial role in ensuring efficient frequency control. Compared to other controllers, the MRAC-FPI-WOA controller proves a significantly higher level of accuracy and responsiveness to ramp variations in solar irradiance levels. Figure 25(b) depicts the output power of the sources. As solar radiation decreases, there is a corresponding increase in the DEG power. Alternatively, as the solar radiation increases, the DEG power also rises to accommodate the changing power consumption needs. To evaluate the efficiency of the system, A comparative analysis is performed on the MRAC-FPI-WOA, PI-WOA, PI-PSO and FPI-WOA controllers. Table 5, along with Fig. 25(c), provides a comprehensive overview of the controlled response of the system frequency under ramp shifts in solar irradiance. The MRAC-FPI-WOA controller ensures stable control of system frequency, even in the face of ramp variations in solar radiation. This proves the controller’s ability to sustain stable performance across different conditions. Moreover, Fig. 25(d) presents additional further by illustrating the voltage levels across the system, further contributing to the general comprehension of the system’s performance.

### Case 5. Random changes in solar radiation

**Fig. 26:**
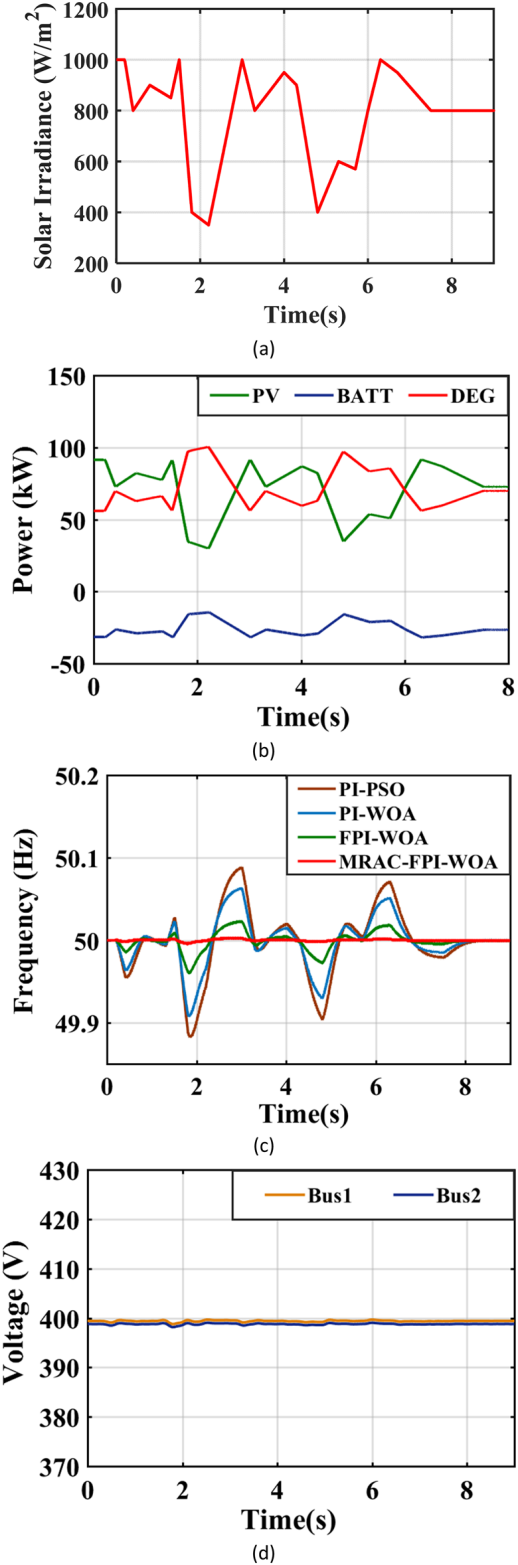
System behavior in case 5. (**a**) Radiation is a random-changed profile, (**b**) Output power of the sources, (**c**) System Frequency, (**d**) System Voltage.

Figure 26(a) shows the erratic behavior of solar irradiance levels over a defined time. In these situations, the MRAC-FPI-WOA controller is crucial for ensuring effective frequency control. When contrasted with other controllers, the MRAC-FPI-WOA controller shows a notably greater degree of precision and responsiveness to unpredictable fluctuations in solar irradiance levels. Figure 26(b) illustrates the output power of the sources. As the intensity of solar irradiance declines, the DEG power grows proportionally. However, when solar radiation increases, the DEG power rises to match the fluctuating energy requirements. Table 5, along with Fig. 26(c), offers an in-depth examination of the system frequency behavior throughout instances of random fluctuations in solar irradiance. The MRAC-FPI-WOA controller successfully maintains the stability of the system frequency, even amidst unpredictable fluctuations in solar irradiance. This highlights the controller’s ability to offer consistent functionality across a range of conditions. Additionally, Fig. 26(d) offers additional insights by illustrating the system’s voltage levels, improving the overall comprehension of system’s operational performance.

### Case 6. A rapid change in load

**Fig. 27:**
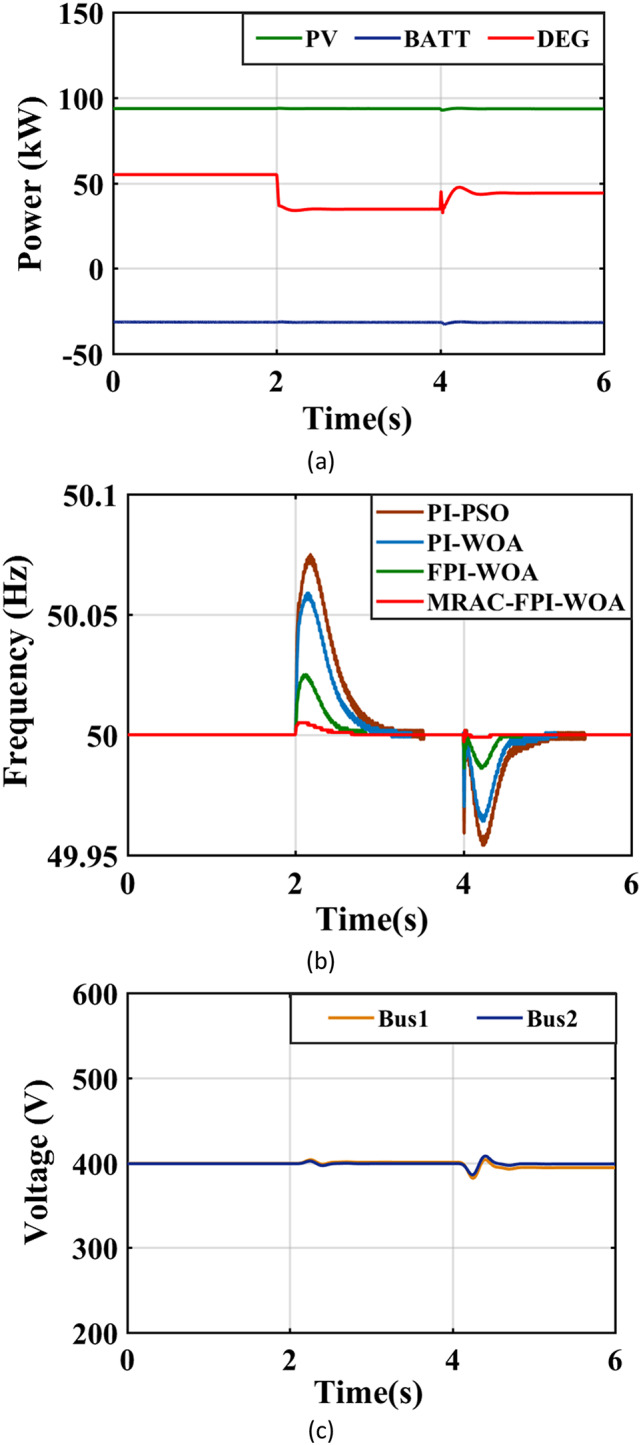
System behavior in case 6. (**a**) Output power of the sources, (**b**) System Frequency, (**c**) System Voltage.

Figure 27(a) illustrates the power output from both the PV and DEG sources during instances of abrupt load changes. At the 2-second mark, there is a notable decrease of 18.3% in the demand for the AC load within the system, dropping from 120 kW to 98 kW. During this period, the power output from the PV systems remains unchanged at 94 kW, while the output from the DEG declines from 56 kW to 35 kW. At the 4-second mark, as depicted in Fig. 27(a), there is an increase of 11.2% in the AC load demand, rising from 98 kW to 109 kW. Throughout this time, the PV power continues to hold steady at 94 kW, while the DEG power rises from 35 kW to 44 kW to satisfy the additional energy requirements. Figure 27(b) and Table 5 present a detailed comparison of the performance of the MRAC-FPI-WOA, FPI-WOA, PI-WOA, and PI-PSO controllers in managing sudden changes in load, emphasizing the MRAC-FPI-WOA controller’s effectiveness in ensuring effective frequency regulation under these conditions. Furthermore, Fig. 27(c) provides a sequential representation of the system’s voltage measurements, offering additional insights into its operational dynamics.

### Case 7. Different disturbances in the system

**Fig. 28:**
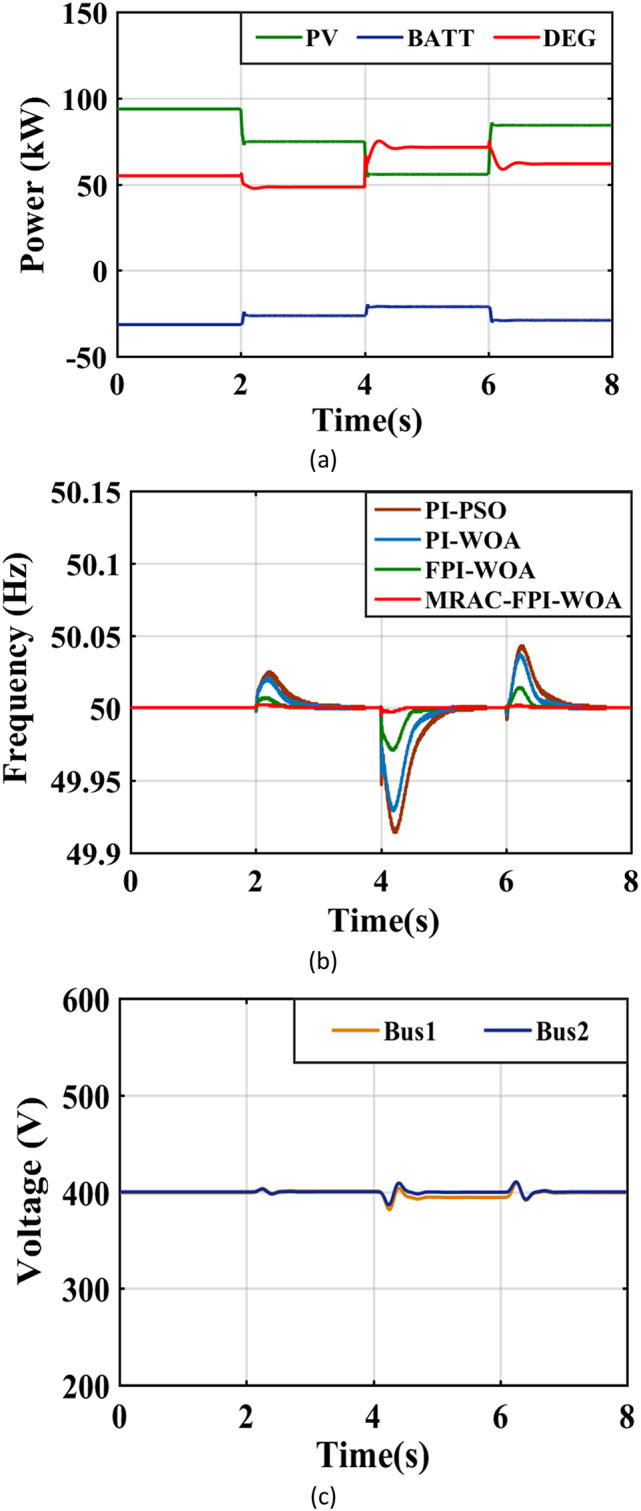
System behavior in case 7. (**a**) Output power of the sources, (**b**) System Frequency, (**c**) System Voltage.

In this case, scenarios (3) and (6) are interconnected and run simultaneously. Figure 28(a) visually depicts the output power of the sources. When solar irradiance reduces from 1000 to 800 W/m² after two seconds, there is also a concurrent 18.3% reduction in the demand for AC load within the system, which drops from 120 kW to 98 kW. At precisely 2 s, the output from the PV systems reduces from 94 kW to 73 kW. This decline in PV power output, combined with the reduced load, leads to a decrease in power generation from the DEG, which falls from 56 kW to about 48 kW to meet the adjusted energy requirements. Subsequently, when solar radiation further decreases to 600 W/m² after an additional four seconds, there is an 11.2% increase in the AC load demand, rising from 98 kW to 109 kW. At this 4-second mark, the PV power drops from 73 kW to 54 kW. In response to this change, DEG’s power generation rises from 48 kW to 72 kW to fulfill the new demand. Additionally, when solar radiation increases from 600 to 900 W/m² at the six-second mark, the AC load demand within the system remains constant at 109 kW. The PV power rises from 54 kW to about 82 kW, resulting in decreased production from the DEG, which reduces from 72 kW to 62 kW. Table 5; Fig. 28(b) provide a comprehensive overview of the system frequency response. The system’s performance efficiency is assessed based on the MRAC-FPI-WOA, PI-WOA, PI-PSO and FPI-WOA controllers, considering several critical parameters, including $\:{\text{T}}_{\text{s}}$, ITAE, %$\:{\text{M}}_{\text{p}}$, and %$\:{\text{M}}_{\text{u}\text{s}}$. Finally, Fig. 28(c) gives deeper insights into displaying the system’s voltage levels.

**Table 5:** The summary of the system behavior under each of the various proposed controllers across different cases.

Cases		Controller type	System Frequency			
			ITAE	$\:{\text{T}}_{\text{s}}$	%$\:{\text{M}}_{\text{u}\text{s}}$	%$\:{\text{M}}_{\text{p}}$
Case 1: Three-phase fault on Bus 2		PI-PSO	3746.361	2.454	1.498	1.104
		PI-WOA	3193.172	2.045	1.156	0.794
		FPI-WOA	1847.275	1.439	0.798	0.706
		MRAC-FPI-WOA	2442.640	1.667	0.407	0.452
Case 2: A three-phase fault happens in the center of the tie line		PI-PSO	4948.759	2.012	1.492	1.124
		PI-WOA	4095.999	1.791	1.154	0.728
		FPI-WOA	2268.751	1.595	0.796	0.698
		MRAC-FPI-WOA	1085.582	1.189	0.308	0.478
Case 3: Step changes in solar radiation	When solar irradiance varies from $\:1000\:(\text{W}/{\text{m}}^{2})\:\text{t}\text{o}\:800\:(\text{W}/{\text{m}}^{2}$)	PI-PSO	471.384	1.995	0.102	0.002
		PI-WOA	313.947	1.975	0.085	0.002
		FPI-WOA	121.237	1.963	0.034	0.002
		MRAC-FPI-WOA	81.883	0.496	0.006	0
	When solar irradiance varies from $\:800\:(\text{W}/{\text{m}}^{2})\:\text{t}\text{o}\:600\:(\text{W}/{\text{m}}^{2}$)	PI-PSO	988.614	1.991	0.101	0.002
		PI-WOA	715.831	1.983	0.087	0.002
		FPI-WOA	259.659	1.958	0.032	0
		MRAC-FPI-WOA	104.609	0.437	0.006	0
	When solar radiation changes from $\:600\:(\text{W}/{\text{m}}^{2})\:\text{t}\text{o}\:900\:(\text{W}/{\text{m}}^{2}$)	PI-PSO	1792.06	1.902	0.002	0.144
		PI-WOA	1475.90	1.732	0.002	0.129
		FPI-WOA	524.781	1.655	0.002	0.052
		MRAC-FPI-WOA	139.895	0.305	0	0.006
Case 4: Ramp changes in solar radiation		PI-PSO	4763.77	8.452	0,042	0.061
		PI-WOA	4127.02	8.165	0.026	0.046
		FPI-WOA	1320.08	8.074	0.008	0.014
		MRAC-FPI-WOA	491.602	6.526	0.002	0.002
Case 5: Random changes in solar radiation		PI-PSO	17389.2	8.364	0.236	0.178
		PI-WOA	13727.7	8.123	0.140	0.130
		FPI-WOA	4959.20	7.661	0.08	0.046
		MRAC-FPI-WOA	829.765	6.518	0.001	0.006
Case 6: A rapid change in load	Reduce the load by 18.3% from 120 kW to 98 kW	PI-PSO	603.729	1.533	0.005	0.151
		PI-WOA	429.345	1.382	0.004	0.123
		FPI-WOA	165.945	0.853	0.002	0.050
		MRAC-FPI-WOA	102.208	0.687	0	0.010
	Raise the load by 11.2% from 98 kW to 109 kW	PI-PSO	916.831	1.454	0.092	0.003
		PI-WOA	746.294	1.113	0.072	0.002
		FPI-WOA	272.501	0.696	0.030	0.002
		MRAC-FPI-WOA	121.686	0.331	0.006	0.002
Case 7: Different disturbances in the system	When solar irradiance varies from 1000 (W/$\:{\text{m}}^{2}$) to 800 (W/$\:{\text{m}}^{2}$), and the load reduces by 18.3% from 120 kW to 98 kW	PI-PSO	311.528	1.749	0.006	0.053
		PI-WOA	195.166	1.524	0.006	0.042
		FPI-WOA	79.4962	0.783	0.004	0.014
		MRAC-FPI-WOA	39.1216	0.528	0	0.004
	When solar irradiance varies from 800 (W/$\:{\text{m}}^{2}$) to 600 (W/$\:{\text{m}}^{2}$) and the load rises by 11.2% from 98 kW to 109 kW	PI-PSO	1027.81	1.688	0.165	0.002
		PI-WOA	819.452	1.183	0.144	0.002
		FPI-WOA	311.425	0.916	0.058	0
		MRAC-FPI-WOA	121.195	0.417	0.010	0
	When solar radiation changes from 600 (W/$\:{\text{m}}^{2}$) to 900 (W/$\:{\text{m}}^{2}$), and the load stays at 109 kW	PI-PSO	1541.95	1.594	0.021	0.087
		PI-WOA	1267.61	1.235	0.010	0.074
		FPI-WOA	460.548	0.606	0.004	0.028
		MRAC-FPI-WOA	148.924	0.423	0	0.004

## Conclusions

This study proposes a robust technique for controlling the frequency of an IHPS, utilizing MRAC-FPI-WOA, FPI-WOA, PI-WOA, and PI-PSO controllers to maintain system stability amid disturbances. The findings highlight the substantial benefits of the MRAC-FPI-WOA controller compared to the FPI-WOA, PI-WOA, and PI-PSO controllers across multiple scenarios. For instance, in Case 1, during a three-phase fault for 100 ms at Bus2, the MRAC-FPI-WOA controller lowers %$\:{\text{M}}_{\text{p}}$ by 59.05%, %$\:{\text{M}}_{\text{u}\text{s}}$ by 72.83%, $\:{\text{T}}_{\text{s}}$ by 32.07%, and ITAE by 34.81% compared to the PI-PSO controller. In Case 2, with a three-phase fault at the tie-line lasting 110 ms, similar improvements are observed, including lowering %$\:{\text{M}}_{\text{p}}$ by 57.47%, %$\:{\text{M}}_{\text{u}\text{s}}\:$by 79.36%, $\:{\text{T}}_{\text{s}}$ by 40.9%, and ITAE by 78.08%, reinforcing the MRAC-FPI-WOA controller’s superior performance in dynamic situations when compared to the PI-PSO controller. In Case 3, MRAC-FPI-WOA showcases its superior adaptability under varying solar irradiance conditions. When irradiance drops from 1000 to 800 W/m², the controller significantly enhances performance by reducing overshoot by 100%, undershoot by 94.12%, settling time by 75.14%, and ITAE by 82.8%. A further decrease from 800 to 600 W/m² yields even better results, undershoot improved by 94.06%, overshoot cut by 100%, settling time improved by 78.05%, and ITAE reduced by 89.47%. Conversely, when solar radiation increases from 600 to 900 W/m², MRAC-FPI-WOA maintains strong performance, decreasing overshoot by 95.38%, undershoot by 100%, settling time by 83.96%, and ITAE by 92.24%. Furthermore, the MRAC-FPI-WOA controller proves improved dynamic responsiveness to ramp changes in solar radiation in Case 4, achieving reductions in %$\:{\text{M}}_{\text{p}}$, %$\:{\text{M}}_{\text{u}\text{s}}$, $\:{\text{T}}_{\text{s}}$, and ITAE by 96.72%, 95.24%, 22.79%, and 89.69%, respectively. In addition, it also shows enhanced adaptability to random fluctuations in solar radiation in Case 5, consistently lowering %$\:{\text{M}}_{\text{p}}$, %$\:{\text{M}}_{\text{u}\text{s}}$, $\:{\text{T}}_{\text{s}}$, and ITAE by 96.63%, 99.58%, 22.07%, and 95.23%, respectively. The MRAC-FPI-WOA controller also proves effective during load variations in Case 6, significantly improving dynamic performance when the load decreases by 18.3% from 120 kW to 98 kW, with reductions in %$\:{\text{M}}_{\text{p}}$ by 93.38%, %$\:{\text{M}}_{\text{u}\text{s}}$ by 100%, $\:{\text{T}}_{\text{s}}$ by 55.19%, and ITAE by 83.08%. Likewise, with a load increase of 11.2% from 98 kW to 109 kW, the MRAC-FPI-WOA controller enhances performance by cutting %$\:{\text{M}}_{\text{p}}$ by 33.33%, %$\:{\text{M}}_{\text{u}\text{s}}$ by 93.48%, $\:{\text{T}}_{\text{s}}$ by 77.24%, and ITAE by 86.79%. In Case 7, MRAC-FPI-WOA exhibits exceptional adaptability under varying operating conditions: when solar irradiance decreases from 1000 to 800 W/m² alongside an 18.3% load reduction (120 kW to 98 kW), it reduces overshoot by 92.45%, undershoot by 100%, settling time by 69.81%, and ITAE by 87.46%; during a further irradiance drop to 600 W/m² with an 11.2% load increase (98 kW to 109 kW), it achieves even better performance with 100% overshoot reduction, 93.94% undershoot reduction, 75.3% settling time improvement, and 88.22% ITAE reduction; and finally, when irradiance rebounds to 900 W/m² at a steady 109 kW load, it maintains superior control with 95.4% overshoot reduction, 100% undershoot suppression, 72.9% faster settling, and 90.4% lower ITAE, demonstrating consistent excellence across all test scenarios. The simulation results confirm that the MRAC-FPI-WOA controller effectively sustains system stability and quality by balancing generation and consumption across diverse operating conditions. While the current study demonstrates the controller’s effectiveness through comprehensive MATLAB/Simulink simulations, we acknowledge that real-time hardware validation, such as HIL (Hardware-in-the-Loop) and Processor-in-the-Loop (PIL) validation, would be necessary to fully verify its performance in practical implementations. Future work will focus on experimental validation using microgrid testbeds with actual power electronics interfaces, robustness testing under real-world communication delays and measurement noise, and comparative analysis with physical benchmark controllers. Additionally, we plan to integrate advanced control techniques, such as machine learning, to further improve adaptability and explore hybrid energy systems that incorporate additional renewable sources, with parallel development of hardware prototypes for field testing.

## Data Availability

All data generated or analyzed during this study are included in this published article.

## Abbreviations

ABC: Artificial bee colony
AC: Alternating current
AFOPI: Adaptive fractional order PI
a-SCA: advanced sine cosine algorithm
BATT: Battery storage
BA: Bat algorithm
BDGOA: Bio-dynamic grasshopper optimization algorithm
COA: Coati optimization algorithm
$\:{\mathbf{C}\mathbf{P}\mathbf{D}}^{\varvec{\upmu\:}}$F − TI: Cascaded tilted-FO derivative with filter
CQOSHO: Chaos quasi-oppositional SHO
CSA: Crow-search algorithm
DC: Direct current
DCSA: Diligent crow search algorithm
DEG: Diesel engine generator
DSA: Dragonfly search algorithm
FFOPID: Fuzzy-fractional order PID
FLC Fuzzy: Logic Controller
MRAC-FPI-WOA: Model reference adaptive control-fuzzy proportional integral based whale optimization algorithm
PID-T: Proportional-integral-derivative-Tilt
PLE: Piecewise Linear-Elliptic
PSO: Particle swarm optimization
P&O: Perturb and observe
PV: Photovoltaic
RES: Renewable energy sources
SHO: Sea-horse optimization
SOC: State of Charge
T2F-CPIF: Type-2 Fuzzy Cascade
TSA: Tunicate search algorithm
TFOPID: Tilt fractional order PID
WOA: Whale optimization algorithm
$\:\text{Α}$: P-N junction ideality factor
E: Solar irradiance
$\:{\text{C}}_{\text{R}}$: Battery rated capacity
$\:{\text{I}}_{sc}$: Short-circuit current
$\:{\text{I}}_{\text{R}}$: Battery internal reaction current
$\:{\text{I}}_{\text{G}}$: Parasitic gassing current
$$\:{\text{I}}_{\text{B}\text{A}\text{T}\text{T}}$$: Battery current
$\:{\text{I}}_{\text{r}\text{s}}$: PV cell’s Reverse leakage current
FOPID: Fractional-Order PID
FOPI: Fractional-Order PI
FPI: Fuzzy Proportional-Integral
GA: Genetic Algorithm
GWO: Grey Wolf Optimizer
HAALO: Hybrid Adaptive Ant Lion Optimization
FOPIλ: Fractional-Order Proportional-Integral
IHPS: Islanded Hybrid Power System
I-SSO: Improved Salp Swarm Optimization
MPC: Model Predictive Control
MPPT: Maximum Power Point Tracking
MPP: Maximum Power Point
MRAC: Model Reference Adaptive Control
OSHO: Opposition-based SHO
PEMFC: Proton Exchange Membrane Fuel Cells
PI: Proportional-Integral
PID: Proportional-Integral-Derivative
$\:{\text{I}}_{out}$: PV cell’s output current
$$\:{\text{I}}_{\text{p}\text{h}}$$: Photocurrent source
K: Boltzmann constant
$\:{\text{k}}_{\text{i}}$: Short-circuit current coefficient
$\:{\text{N}}_{\text{S}}$: Number of PV cells connected in series
$\:{\text{N}}_{\text{P}}$: Number of PV cells arranged in parallel
$$\:{\text{P}}_{\text{L}\text{o}\text{a}\text{d}}$$: Load power
$\:{\text{P}}_{\text{D}\text{E}\text{G}}$: Diesel engine generator power
$$\:{\text{P}}_{\text{P}\text{V}}$$: Photovoltaic power
q: Electron charge
$$\:{\text{R}}_{\text{s}\text{e}\text{r}}$$: Series resistance
$$\:{\text{R}}_{\text{s}\text{h}}$$: Shunt resistance
T: P-N junction temperature
$\:{\text{T}}_{\text{B}\text{A}\text{T}\text{T}}$: Battery operating temperature
$\:{\text{T}}_{\text{r}}$: Cell reference temperature
$$\:{\text{T}}_{\text{s}}$$: Settling time
$\:{\text{V}}_{\text{B}\text{A}\text{T}\text{T}}$: Battery terminal voltage
$\:{\text{V}}_{\text{o}\text{u}\text{t}}$: PV cell’s terminal voltage
$\:{\text{V}}_{oc}$: Open-circuit voltage
%$$\:{\text{M}}_{\text{p}}$$: Maximum overshoot
%$$\:{\text{M}}_{\text{u}\text{s}}$$: Maximum undershoot
